# Mn^2+^ modulates the expression of cellulase genes in *Trichoderma reesei* Rut-C30 via calcium signaling

**DOI:** 10.1186/s13068-018-1055-6

**Published:** 2018-03-01

**Authors:** Yumeng Chen, Yaling Shen, Wei Wang, Dongzhi Wei

**Affiliations:** State Key Lab of Bioreactor Engineering, New World Institute of Biotechnology, East China University of Science and Technology, East China University of Science and Technology, 130 Meilong Road, P.O.B. 311, Shanghai, 200237 China

**Keywords:** *Trichoderma reesei*, Mn^2+^ stimulation, Cellulase, Mn^2+^ transport, Calcium signaling, TPMR1

## Abstract

**Background:**

The filamentous fungus *Trichoderma reesei* Rut-C30 is one of the most vital fungi for the production of cellulases, which can be used for biofuel production from lignocellulose. Nevertheless, the mechanism of transmission of external stimuli and signals in modulating cellulase production in *T. reesei* Rut-C30 remains unclear. Calcium is a known second messenger regulating cellulase gene expression in *T. reesei*.

**Results:**

In this study, we found that a biologically relevant extracellular Mn^2+^ concentration markedly stimulates cellulase production, total protein secretion, and the intracellular Mn^2+^ concentration of Rut-C30, a cellulase hyper-producing strain of *T. reesei*. Furthermore, we identified two Mn^2+^ transport proteins, designated as TPHO84-1 and TPHO84-2, indicating that they are upstream in the signaling pathway that leads to cellulase upregulation. We also found that Mn^2+^ induced a significant increase in cytosolic Ca^2+^ concentration, and that this increased cytosolic Ca^2+^ might be a key step in the Mn^2+^-mediated regulation of cellulase gene transcription and production. The utilization of LaCl_3_ to block plasma membrane Ca^2+^ channels, and deletion of *crz1* (calcineurin-responsive zinc finger transcription factor 1) to interrupt calcium signaling, showed that Mn^2+^ exerts the induction of cellulase genes via calcium channels and calcium signaling. To substantiate this, we identified a Ca^2+^/Mn^2+^ P-type ATPase, TPMR1, which could play a pivotal role in Ca^2+^/Mn^2+^ homeostasis and Mn^2+^ induction of cellulase genes in *T. reesei* Rut-C30.

**Conclusions:**

Taken together, our results revealed for the first time that Mn^2+^ stimulates cellulase production, and demonstrates that Mn^2+^ upregulates cellulase genes via calcium channels and calcium signaling. Our research also provides a direction to facilitate enhanced cellulase production by *T. reesei*.

**Electronic supplementary material:**

The online version of this article (10.1186/s13068-018-1055-6) contains supplementary material, which is available to authorized users.

## Background

Lignocellulosic biomass, the most abundant renewable energy source, can be hydrolyzed to sugars for bioethanol production. A common host cited for the production of cellulases and hemicellulases is the saprotrophic, filamentous fungus *Trichoderma reesei*, which is well known for its excellent ability to secrete a broad range of cellulases at very high levels [1–4]. Due to its ability to degrade and thrive on cellulose-containing fabrics, *T. reesei* has attracted attention and was consequently studied in detail [5, 6]. However, compared with the energy-efficient production of cellulases and hemicellulases, the induction and regulation of the expression of genes enoding these enzymes in *T. reesei* are still not completely understood. Additionally, the induction of high-level cellulase production is dependent on inducers such as cellulose, d-xylose, lactose, or sophorose [3, 7–9], increasing the costs for the application of produced enzymes. Due to the extensive applications of cellulases and hemicellulases, the induction and regulation of the expression of genes encoding these enzymes have drawn significant attention.

Recent studies have demonstrated that cellulase production is regulated in response to environmental stress, such as light [10, 11], organic solvents [12], and metal ions [13–15]. A great example was shown by Chen and co-workers, who illustrated that Ca^2+^ plays an important role in the production of cellulase or hemicellulase in *T. reesei* Rut-C30 [13]. Chen et al. [13] suggested that external Ca^2+^ stimulated hyphal growth, growth-independent cellulase production, and total protein secretion of *T. reesei* Rut-C30 through the Ca^2+^ (/calmodulin)–calcineurin–CRZ1 signal transduction pathway.

Intracellular accumulation of Mn^2+^ can interfere with calcium metabolism [16]. In *Ganoderma lucidum*, one of the most well-known medicinal basidiomycetes producing many bioactive compounds such as ganoderic acids, Mn^2+^ is thought to enhance cytosolic Ca^2+^ to induce ganoderic acid biosynthesis through the calcineurin signal pathway, to upregulate its biosynthetic genes at the transcriptional level [17]. In *Aspergillus nidulans*, high levels of Mn^2+^ can induce an increase in intracellular Ca^2+^ levels, which leads to the nuclear accumulation of CrzA [18, 19]. These observations suggest that Ca^2+^ and Mn^2+^ have relevant impacts on the cellular physiology and metabolism of various organisms.

The ability to sense and respond to Mn^2+^ by the production of import and efflux systems to maintain Mn^2+^ homeostasis is critical for cells [20–23]. Such homeostasis factors include cell surface and intracellular Mn^2+^ transporters that collectively guide the metal through a designated trafficking pathway [20, 22]. PMR1, a P-type ATPase ion pump, is a transporter for both Ca^2+^ and Mn^2+^ and is also a homeostasis factor, associating with delivering both Mn^2+^ and Ca^2+^ to the secretory pathway [22, 24–26]. Cytosolic Mn^2+^ accumulates in yeast cells lacking the PMR1 transporter [22]. Although many studies have reported on Mn^2+^ homeostasis in various organisms, the detailed mechanisms are yet unclear. First, studies have focused mainly on yeast or bacterial species, and less work has been conducted with filamentous fungi. Second, the detailed roles of Mn^2+^ in the biological processes of filamentous fungi remain unclear and need further studies. Third, the conjunction between Mn^2+^ and calcium signaling in filamentous fungi is still not clear. Therefore, it is necessary to study the mechanism of Mn^2+^ stimulation in filamentous fungi.

In this study, the impact of Mn^2+^ on the growth and protein production of *T. reesei* Rut-C30 was investigated. The temporal dynamics of intracellular and extracellular Mn^2+^ were detected. Additionally, the function of Mn^2+^ transport proteins in *T. reesei* Rut-C30 was characterized. The conjunction between Mn^2+^ and Ca^2+^ was further investigated to elucidate how Mn^2+^ regulates the production of cellulase via calcium signaling in *T. reesei* Rut-C30. These results could be used for more efficient production of cellulase by *T. reesei*, and provide a new approach to understand the regulatory mechanisms that respond to environmental stimuli. This research may also offer the basis for the study of Mn^2+^-induced signal transduction in other fungi.

## Results

### Effects of the addition of Mn^2+^ on growth and cellulase production of *T. reesei*

**Fig. 1:**
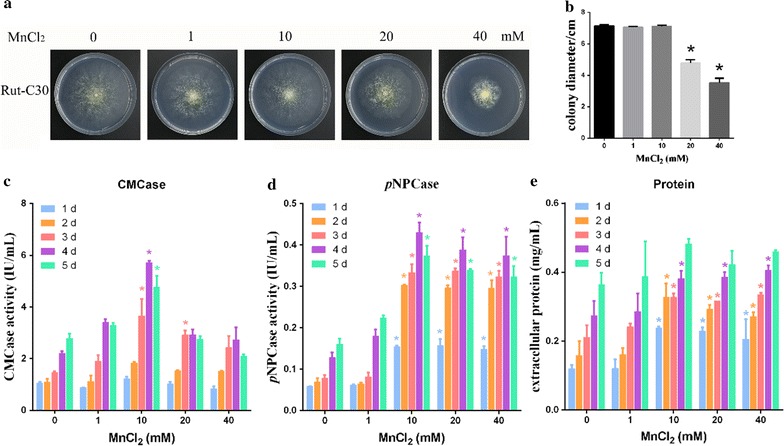
Effects of Mn^2+^ on hyphal growth and protein production in *T. reesei* Rut-C30. **a** The hyphal growth of *T. reesei* Rut-C30 on plates. Mn^2+^ was added at a final concentration of 1, 10, 20, or 40 mM. **b** The measurement of colony diameter. The effects of different concentrations of Mn^2+^ (final concentration 0, 1, 10, 20, and 40 mM) on CMCase activity (**c**), *p*NPCase activity (**d**), and total protein concentrations (**e**) of *T. reesei* Rut-C30. Values are the mean ± SD of the results from three independent experiments. Asterisks indicate a significant difference compared to the untreated strain (*p < 0.05, Student’s *t* test)

To determine how Mn^2+^ influences the hyphal growth, *T. reesei* Rut-C30 strains were cultured on MM (minimal medium) plates supplemented with different concentrations of Mn^2+^ (0, 1, 10, 20, and 40 mM final concentration) and 2% glucose as the sole carbon source. The mycelium length of *T. reesei* Rut-C30 after addition Mn^2+^ is shown in Fig. 1a. There was no significant difference in the hyphal growth with 1–10 mM Mn^2+^. However, when the concentrations of Mn^2+^ increased to 20 mM, the strains grew more slowly and sparsely. As shown in Fig. 1b, treatment with 20 mM Mn^2+^ caused a 30% reduction in the colony diameter compared with that of the untreated strains. Moreover, after treatment with 40 mM Mn^2+^, the treated strains showed a severe reduction in the colony diameter (53%).

To evaluate the effects of Mn^2+^ on cellulase production and total protein secretion, the same weight of precultured mycelia of *T. reesei* Rut-C30 was transferred to liquid MM containing 1% Avicel as the sole carbon source and different concentrations of Mn^2+^ (0, 1, 10, 20 and 40 mM). As shown in Fig. 1c (Additional file 8: Figure S7A), addition of Mn^2+^ at a final concentration of 10 mM significantly stimulated CMCase activity (representing *endo*-β-glucanase activity), with an increase of approximately 140% compared to the controls without addition of Mn^2+^. However, 1, 20, and 40 mM of Mn^2+^ did not evidently affect CMCase activity. As shown in Fig. 1d (Additional file 8: Figure S7B) and e, the addition of Mn^2+^ at a final concentration of 10–40 mM significantly stimulated *p*NPCase activity (representing *exo*-β-glucanase activity) and increased total protein concentration after 1 day of fermentation, with an increase of approximately 327 and 55%, respectively.

The above results demonstrated that 10–40 mM Mn^2+^ could stimulate cellulase production and total protein secretion in *T. reesei* Rut-C30, and that 20–40 mM Mn^2+^ could delay hyphal growth. The optimal concentration of Mn^2+^ to enhance cellulase production was 10 mM, which was selected for further research in our study. CMCase or *p*NPCase activity was directly used to represent cellulase activity in our study.

To further determine the effects of Mn^2+^ supplementation on the synthesis of cellulases or total protein secretion, the expression levels of four main cellulase genes (*cbh1* encoding cellobiohydrolase I, *cbh2* encoding cellobiohydrolase II, *egl1* encoding endoglucanase I, and *egl2* encoding endoglucanase II), and a transcriptional regulator of cellulases, *xyr1,* were compared by quantitative reverse-transcription PCR (RT-qPCR) after induction for 24, 48, and 72 h in cultures with 0 or 10 mM Mn^2+^ supplementation. The primers used to detect transcriptional changes of these genes are listed in Additional file 1: Table S1. The transcriptional levels of four main cellulase genes significantly increased by almost 2- to 3.5-fold, after 24, 48, and 72 h of induction following supplementation with 10 mM Mn^2+^ (see Additional file 2: Figure S1A–D). In *T. reesei*, XYR1 is a global transcriptional activator of cellulose and hemicellulase genes [27]. In accordance with the transcription of *cbh1*, *cbh2* and *egl1*, *egl2*, the expression level of *xyr1* was also significantly stimulated after 72 h of induction (see Additional file 2: Figure S1E). These results were consistent with the upregulation of cellulase activity through the addition of 10 mM Mn^2+^. However, when compared to expression of other cellulase-related genes, the delayed upregulation of *xyr1* expression implied that other putative regulators may participate in Mn^2+^ metabolism/regulation to directly induce cellulase gene expression, besides indirect induction through *xyr1*.

### Variation in intracellular and extracellular Mn^2+^ concentration after the addition of Mn^2+^

**Fig. 2:**
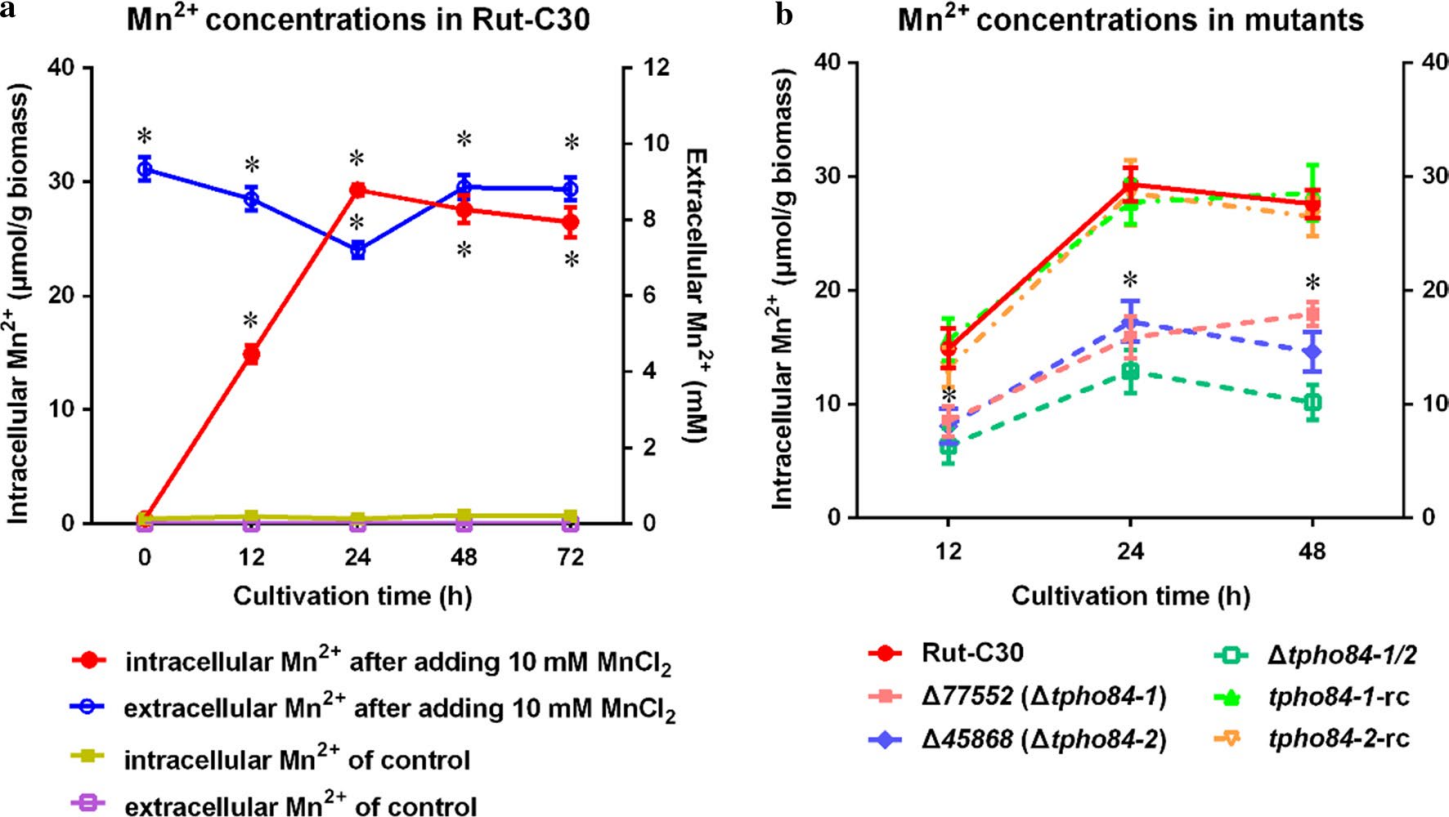
Concentrations of Mn^2+^ in *T. reesei* Rut-C30 and its derivative mutants. **a** Filled circle, intracellular Mn^2+^ concentration after adding 10 mM MnCl_2_; blank circle, extracellular Mn^2+^ concentration after adding 10 mM MnCl_2_; filled square, intracellular Mn^2+^ concentration of control without MnCl_2_ supplement; blank square, extracellular Mn^2+^ concentration of control without MnCl_2_ supplement. **b** The concentrations of intracellular Mn^2+^ of *T. reesei* Rut-C30 and its derivative mutant strains were examined after cultured in medium containing 10 mM MnCl_2_. Values are the mean ± SD of the results from three independent experiments. Asterisks indicate significant differences from parental strain Rut-C30 (*p < 0.05, Student’s *t* test)

Extracellular Mn^2+^ can significantly augment the intracellular Mn^2+^ content, and subsequently affect the physiology and metabolism of *G. lucidum* [17]. To investigate whether enhanced cellulase production is linked to intracellular Mn^2+^ in *T. reesei* Rut-C30, the intracellular and extracellular Mn^2+^ concentrations were measured by inductively coupled plasma mass spectrometry (ICP-MS) during cultivation. As illustrated in Fig. 2a, the levels of intracellular and extracellular Mn^2+^ were almost constant in the control sample without Mn^2+^ addition. On the contrary, upon Mn^2+^ addition (10 mM final concentration), the intracellular Mn^2+^ concentration initially markedly increased, reaching its maximum at 24 h and then declined gradually, while the extracellular Mn^2+^ concentration dropped initially, reaching its minimum at 24 h and then increased gradually, suggesting that Mn^2+^ was transported into cells initially in response to a higher extracellular Mn^2+^ concentration. Subsequently, intracellular Mn^2+^ gradually effused into the medium from 24 h. We hypothesized that a mechanism could pump Mn^2+^ in and out of the cell.

### Identification of Mn^2+^ transport proteins TPHO84-1 and TPHO84-2

Jensen et al. [16] suggested that PHO84, a low-affinity transporter of Mn^2+^ from *Saccharomyces cerevisiae*, transports Mn^2+^ when cells are exposed to higher Mn^2+^ concentrations. In our study, Mn^2+^ efficiently transported into *T. reesei* Rut-C30 cells at an Mn^2+^ concentration of 10 mM (Fig. 2a). To identify the protein(s) responsible for transporting Mn^2+^ in *T. reesei*, we conducted a homology search with the protein sequence of PHO84 (GenBank: KZV08715.1) in the *T. reesei* genome from the JGI database (http://genome.jgi.doe.gov/Trire2/Trire2.home.html). Five high score hits were obtained with the following protein identity matches: TRE77552, 59.6%, TRE81389, 51.8%, TRE45852, 49.0%, TRE45868, 40.1%, and TRE106118, 35.2%.

We hypothesized that Mn^2+^ transport proteins would be highly expressed during Mn^2+^ addition. To assess which proteins encoded putative Mn^2+^ transport activity, transcriptional levels of *tre77552*, *tre81389*, *tre45852*, *tre45868* and *tre106118* were monitored by RT-qPCR with 10 mM Mn^2+^ compared to no Mn^2+^ addition at different induction times. As illustrated in Additional file 3: Figure S2, expression of these genes was significantly upregulated in the samples treated with 10 mM Mn^2+^. Based on their induction at higher Mn^2+^ concentration, all five genes might be responsible for Mn^2+^ transport of *T. reesei* Rut-C30.

To further investigate whether any of these five proteins displayed a function similar to that of PHO84, involved in transport of Mn^2+^ in *S. cerevisiae*, the five deletion mutants, Δ*77552*, Δ*81389*, Δ*45852*, Δ*45868*, and Δ*106118*, respectively, were collected to measure intracellular Mn^2+^ concentrations by ICP-MS compared to that in the parental strain. As shown in Fig. 2b, Δ*77552* and Δ*45868* showed a marked decrease in steady-state levels of cellular Mn^2+^ when compared with the Rut-C30 strain, in which a 70–80% decrease in metal accumulation was obtained. However, under the same conditions, Δ*81389*, Δ*45852*, or Δ*106118* had an Mn^2+^ concentration similar to that in Rut-C30 strain (see Additional file 4: Figure S3). The *tre77552* and *tre45868* double mutant strain Δ*tpho84*-*1/2* showed lower intracellular Mn^2+^ concentration than each single mutant (Fig. 2b). Δ*77552* and Δ*45868* were also complemented by transforming vectors p*tpho84*-*1*-rc and p*tpho84*-*2*-rc into them, respectively (see Additional file 5: Figure S4). Complementation strains (*tpho84*-*1*-rc and *tpho84*-*2*-rc) were obtained to demonstrate the restoration of Mn^2+^ transport with intracellular Mn^2+^ concentrations similar to those of the parent strain Rut-C30 (Fig. 2b). The results demonstrated that the intracellular Mn^2+^ concentration can be increased via putative Mn^2+^ transport proteins TRE77552 and TRE45868 in *T. reesei* Rut-C30.

As predicted by SMART (http://smart.embl-heidelberg.de/), both TRE77552 and TRE45868 are membrane proteins with an 11- and 9-transmembrane domain topology, respectively. To visualize the location of TRE77552 and TRE45868, we constructed two chimeric proteins, RFP-77552 and RFP-45868, by fusing red fluorescence protein to their N-terminus (see Additional file 6: Figure S5A). The two chimeric proteins were overexpressed via *cbh1* promoter, which allowed us to confirm that TRE77552 and TRE45868 are located at the mycelial surface. Additional file 6: Figure S5B shows the in vivo epifluorescence analysis of the two chimeric protein (RFP-77552 and RFP-45868) transformants, rfp-*tpho84*-*1* and rfp-*tpho84*-*2*, respectively, depicting strong and stable fluorescent signal at the mycelial surface, as expected for plasma membrane proteins. However, overexpression of genes encoding membrane bound proteins by a strong promoter might cause some uncertainty.

Based on these findings, we considered that both *tre77552* and *tre45868* encode Mn^2+^ transports with Mn^2+^ transport function located at the plasma membrane, and named the two genes as *tpho84*-*1* and *tpho84*-*2*, respectively. Intracellular Mn^2+^ can be transported into *T. reesei* Rut-C30 cells via TPHO84-1 and TPHO84-2. It is presently unclear whether *tre81389*, *tre45852*, and *tre106118* are inactive in the transport of Mn^2+^.

### Role of TPHO84-1 and TPHO84-2 in cellulase production

**Fig. 3:**
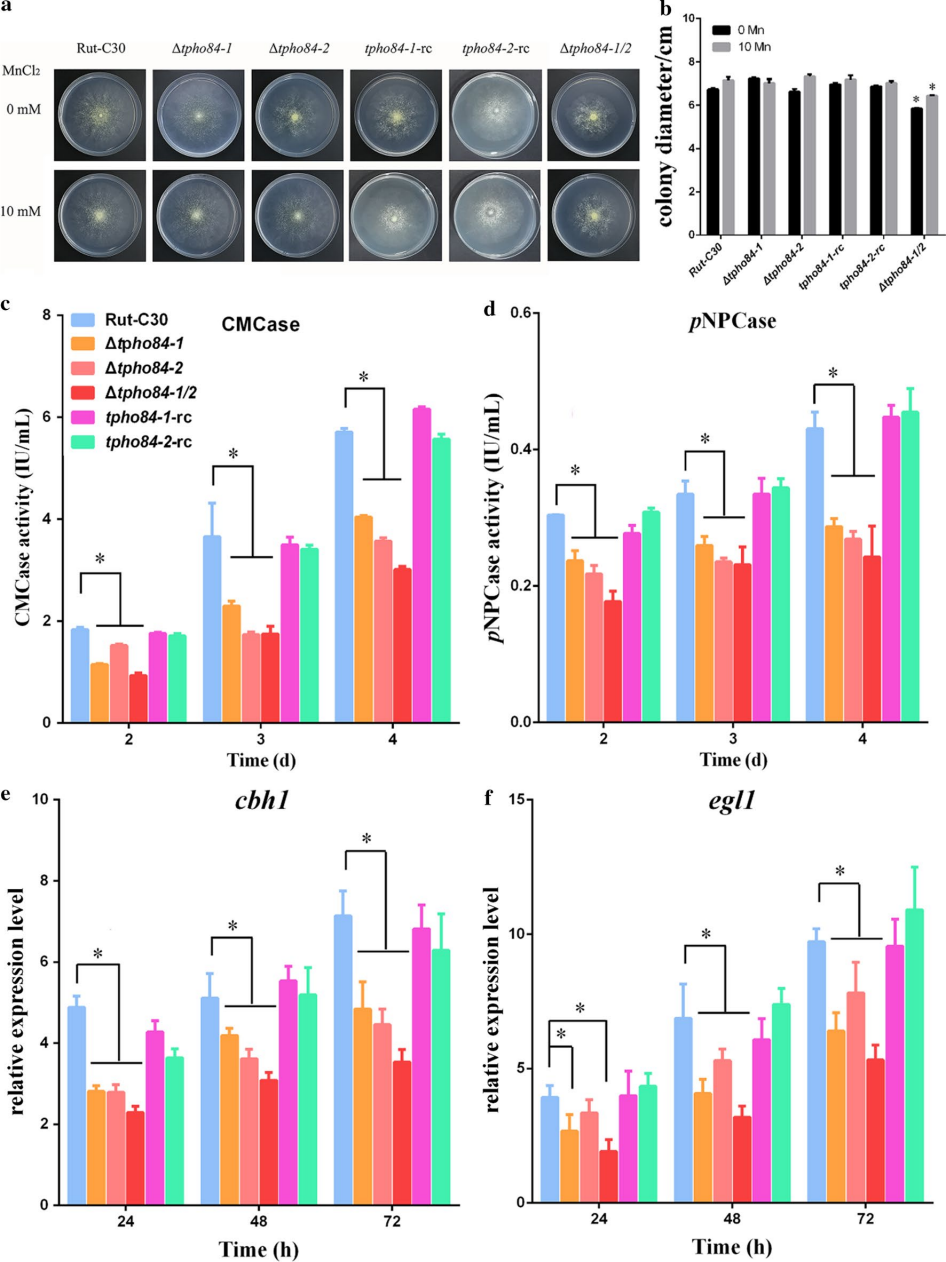
TPHO84-1 and TPHO84-2 regulate cellulase production of *T. reesei* Rut-C30 with Mn^2+^ supplementation. **a** The hyphal growth of *T. reesei* Rut-C30 and its derivative mutant strains under 0 or 10 mM MnCl_2_. **b** The measurement of colony diameter. The CMCase activity (**c**) and *p*NPCase activity (**d**) of *T. reesei* Rut-C30 and its derivative mutant strains were examined after culture in medium containing 0 or 10 mM MnCl_2_. The transcriptional levels of *cbh1* (**e**) and *egl1* (**f**) in the parental strain Rut-C30 and its derivative mutant strains were analyzed after cultured in medium containing 0 or 10 mM MnCl_2_. Values are the mean ± SD of the results from three independent experiments. Asterisks indicate significant differences (*p < 0.05, Student’s *t* test)

Δ*77552* (Δ*tpho84*-*1*) and Δ*45868* (Δ*tpho84*-*2*), and their complementation strains *tpho84*-*1*-rc and *tpho84*-*2*-rc were used to determine the effect of TPHO84-1 and TPHO84-2 in mediating the growth and cellulase production in *T. reesei* Rut-C30. To determine the effect of TPHO84-1 and TPHO84-2 on mediating growth, Rut-C30, Δ*tpho84*-*1*, Δ*tpho84*-*2*, *tpho84*-*1*-rc, *tpho84*-*2*-rc and Δ*tpho84*-*1/2* strains were cultured on minimal medium plates adding 0 or 10 mM Mn^2+^ and 2% glucose as the sole carbon source. The mycelium morphology is shown in Fig. 3a. There was no significant effect on the hyphal growth of Rut-C30, Δ*tpho84*-*1*, Δ*tpho84*-*2*, *tpho84*-*1*-rc and *tpho84*-*2*-rc strains at 0 or 10 mM Mn^2+^. However, the hyphal growth of the Δ*tpho84*-*1/2* transformant was slightly repressed, compared with that of the parent strain at both 0 and 10 mM Mn^2+^ (Fig. 3b).

To further test the effect of Mn^2+^ transport proteins on cellulase production, we investigated the effect of Rut-C30, Δ*tpho84*-*1*, Δ*tpho84*-*2*, *tpho84*-*1*-rc, *tpho84*-*2*-rc, and Δ*tpho84*-*1/2* strains on cellulase production after supplementation of 0 or 10 mM Mn^2+^. There was no obvious difference in CMCase (see Additional file 7: Figure S6A) and *p*NPCase (see Additional file 7: Figure S6B) activities between these six strains without Mn^2+^ addition. However, Δ*tpho84*-*1* and Δ*tpho84*-*2* strains showed approximately 30–40% reduction in CMCase activity, compared with the parental strain Rut-C30 with 10 mM Mn^2+^ addition. Furthermore, Δ*tpho84*-*1/2* strain had lower CMCase activity than each single mutant, at approximately 50% reduction compared with Rut-C30 (Fig. 3c and Additional file 8: Figure S8A). Similarly, Δ*tpho84*-*1* and Δ*tpho84*-*2* strains showed approximately 25 to 30% reduction in the *p*NPCase activity, compared with the parental strain Rut-C30, with 10 mM Mn^2+^ addition. Furthermore, the Δ*tpho84*-*1/2* strain showed lower *p*NPCase activity than each single mutant, and approximately 40% reduction compared with Rut-C30 (Fig. 3d and Additional file 8: Figure S8B). The cellulase production capabilities of *tpho84*-*1*-rc and *tpho84*-*2*-rc were complemented at 10 mM Mn^2+^ supplementation compared to parental strains Δ*tpho84*-*1* and Δ*tpho84*-*2*, respectively, to a level similar to that of the original strain Rut-C30 (Fig. 3c, d and Additional file 8: Figure S8A, B).

Additionally, RT-qPCR was performed to determine the transcription levels of the important cellulase genes *cbh1* and *egl1* in the *T. reesei* Rut-C30, Δ*tpho84*-*1*, Δ*tpho84*-*2*, *tpho84*-*1*-rc, *tpho84*-*2*-rc, and Δ*tpho84*-*1/2* mutants. The primers used to detect transcriptional levels of these genes are listed in Additional file 1: Table S1. In agreement with the CMCase activity levels, no obvious difference in transcription levels of *cbh1* and *egl1* between above six strains was observed without Mn^2+^ addition (data not shown). However, the deletion strains Δ*tpho84*-*1*, Δ*tpho84*-*2*, and Δ*tpho84*-*1/2* showed a marked reduction in cellulase transcription compared with the parental strain Rut-C30 upon 10 mM Mn^2+^ addition at all time points examined (Fig. 3e, f). Similarly, the expression levels of the *cbh1* and *egl1* genes of *tpho84*-*1*-rc and *tpho84*-*2*-rc were complemented under Mn^2+^ supplementation, to a level similar to that of the original strain Rut-C30.

These results indicate that TPHO84-1 and TPHO84-2 participate in inducing cellulase production of *T. reesei* Rut-C30 only under Mn^2+^ addition, which is in accordance with its function as Mn^2+^ transport protein.

### Increase in cytosolic Ca^2+^ level and calcium signaling after Mn^2+^ addition

**Fig. 4:**
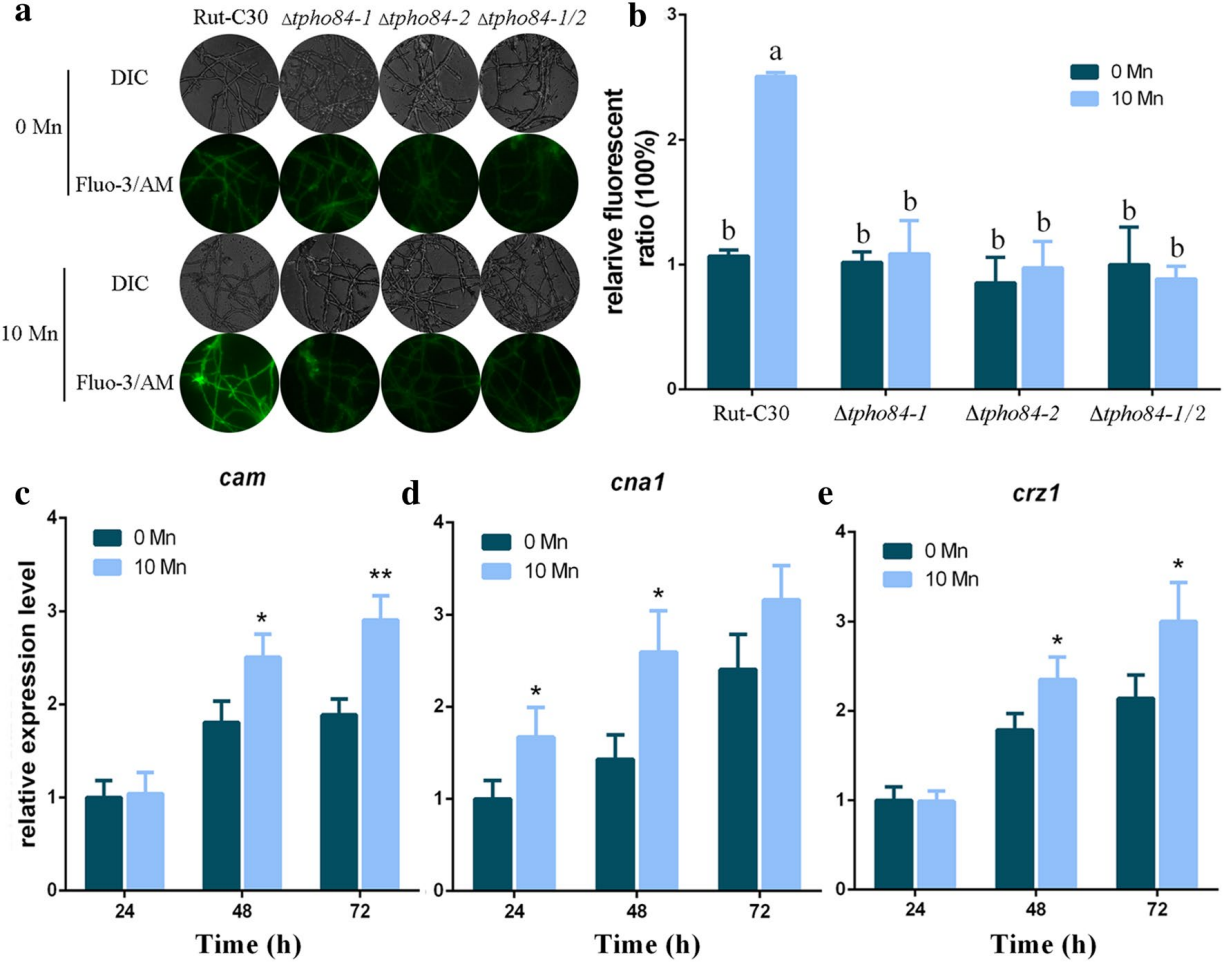
Cytosolic Ca^2+^ levels and calcium signaling increase after Mn^2+^ addition. **A** The analysis of cytosolic Ca^2+^ levels via a Ca^2+^ fluorescent probe Fluo-3/AM. The *T. reesei* Rut-C30 and its derivative mutant strains were cultured in liquid minimal medium for 48–60 h with 0 or 10 mM final concentration of MnCl_2_ (0 or 10 Mn, respectively). For detection, 50 μM Fluo-3/AM was used, and the intensity was monitored using Automatic Inverted Fluorescence Microscopy. Green fluorescence represents the free cytosolic Ca^2+^. DIC, differential interference contrast. **B** Comparative fluorescence ratio analysis of Mn^2+^ influence on cytosolic Ca^2+^ levels. The *y*-axis represents the Ca^2+^ fluorescence ratio measured by CLSM and the *x*-axis the different strains tested. The effects of Mn^2+^ on the expression levels of certain calcium signaling-related genes *cam* (**C**), *cna1* (**D**) and *crz1* (**E**) in *T. reesei* Rut-C30. 0 Mn, no MnCl_2_ was added to the medium; 10 Mn, final concentration of 10 mM MnCl_2_. Values are the mean ± SD of the results from three independent experiments. Asterisks indicate significant differences from untreated strains (*p < 0.05, **p < 0.01, Student’s *t* test). Different letters indicate significant differences between the columns (*p* < 0.05, according to Duncan’s multiple-range test)

Next, we investigated how Mn^2+^ addition can upregulate cellulase gene expression via Mn^2+^ transport proteins TPHO84-1 and TPHO84-2. Using Fluo-3/AM fluorescent dye, a dye that only emits green fluorescence after crossing the cell membrane and binding with Ca^2+^ [28], whose intensity represents relative amounts of free intracellular Ca^2+^ [29], we found that the cytosolic Ca^2+^ concentration was increased after Mn^2+^ addition. As shown in Fig. 4A, a stronger green fluorescence intensity was observed in the Rut-C30 cells under 10 mM Mn^2+^ supplement on the second day than that observed with the control (no Mn^2+^ supplement), demonstrating that Mn^2+^ leads to an increase in the level of cytosolic Ca^2+^. The fluorescence level emitted by the Ca^2+^-activated fluorochrome reached a 2.23-fold increase under 10 mM Mn^2+^ addition compared to that in the control (Fig. 4B). These results indicate that Mn^2+^ induced an increase in the concentration level of cytosolic Ca^2+^. A similar phenomenon was reported in *S. cerevisiae* [30].

We measured the level of cytosolic Ca^2+^ in the Δ*tpho84*-*1*, Δ*tpho84*-*2*, and Δ*tpho84*-*1/2* strains with Mn^2+^ addition. As illustrated in Fig. 4A, B, the significant increase in intracellular Ca^2+^ induced by Mn^2+^, observed in parental strain Rut-C30, was absent in the Δ*tpho84*-*1*, Δ*tpho84*-*2*, and Δ*tpho84*-*1/2* strains. The augmentation of cytosolic Ca^2+^ levels induced by Mn^2+^ was blocked in the Δ*tpho84*-*1*, Δ*tpho84*-*2*, and Δ*tpho84*-*1/2* strains. These results suggest that rising cytosolic Ca^2+^ levels depend on Mn^2+^ transport into Rut-C30 cells via TPHO84-1 and TPHO84-2. This also indicates that Ca^2+^ influx in cells is closely associated with Mn^2+^ homeostasis.

Previous studies have demonstrated that the calcium signal transduction pathway can upregulate cellulase gene expression [13]. We investigated whether the increased levels of cytosolic Ca^2+^, induced by Mn^2+^, can trigger calcium signal transduction pathways in *T. reesei*. To test our hypothesis, RT-qPCR was carried out to analyze the transcriptional levels of calcium signaling-related genes, including calmodulin (*cam*, GenBank: ACZ26150.1), calcineurin (*cna1*, GenBank: EGR49476.1) [31], and calcineurin-responsive zinc finger transcription factor 1, *crz1* [13], under Mn^2+^ addition. The primers used to detect transcription of these genes are listed in Additional file 1: Table S1. As shown in Fig. 4C–E, similar to the increase in the contents of cytosolic Ca^2+^, expression of these genes was significantly upregulated with Mn^2+^ addition. These results suggested that Mn^2+^ can increase the concentration of cytosolic Ca^2+^, thus stimulating the calcium signal transduction pathway to induce cellulase production in *T. reesei* Rut-C30.

#### Mn^2+^ induces cellulase production via cytosolic Ca^2+^

**Fig. 5:**
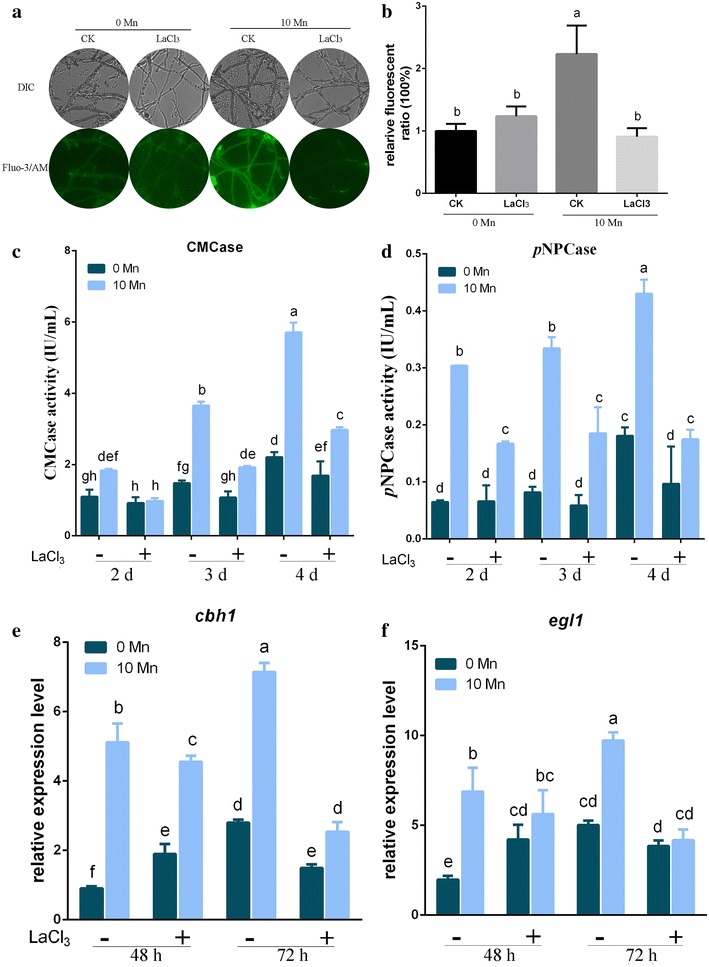
Effects of Ca^2+^ channel inhibitor LaCl_3_ on the cytosolic Ca^2+^ concentration and cellulase production. **A** Fluorescence analysis of LaCl_3_ influence on cytosolic Ca^2+^ burst induced by Mn^2+^. The *T. reesei* Rut-C30 were cultured in liquid minimal medium for 48–60 h with 0 or 10 mM MnCl_2_ (0 or 10 Mn, respectively), and then treated with 0 or 5 mM LaCl_3_. For detection, 50 μM Fluo-3/AM was used, and the intensity was monitored using Automatic Inverted Fluorescence Microscopy. Green fluorescence represents the free cytosolic Ca^2+^. DIC, differential interference contrast, CK, not treated with LaCl_3_. **B** Comparative fluorescence ratio analysis of LaCl_3_ influence on the cytosolic Ca^2+^ burst induced by Mn^2+^. The *y*-axis represents the Ca^2+^ fluorescence ratio measured by CLSM and the *x-*axis the different treatments. The CMCase activity (**C**) and *p*NPCase activity (**D**) of *T. reesei* Rut-C30 were examined after culture in medium containing 0 or 10 mM MnCl_2_ and with (−) or without (+) 5 mM LaCl_3_. The expression levels of *cbh1* (**E**) and *egl1* (**F**) in *T. reesei* Rut-C30 were analyzed after culture in medium containing 0 or 10 mM MnCl_2_ and with (−) or without (+) 5 mM LaCl_3_. Values are the mean ± SD of the results from three independent experiments. Different letters indicate significant differences between the columns (*p* < 0.05, according to Duncan’s multiple-range test)

To investigate the assumption that Mn^2+^ induces cytosolic Ca^2+^ improvement and cellulase production via Ca^2+^ channels, we used LaCl_3_, a plasma membrane Ca^2+^ channel blocker to prevent influx of external Ca^2+^ [32]. Figure 5A, B shows that the Fluo-3/AM fluorescence intensity of mycelia remarkably reduced almost 60% with LaCl_3_ compared with no LaCl_3_ addition, under 10 mM Mn^2+^. The increased content of cytosolic Ca^2+^ induced by Mn^2+^ could be effectively attenuated by adding LaCl_3_ to *T. reesei* Rut-C30. Meanwhile, the increased expression of calcium signaling-related genes, *cam*, *cna1*, and *crz1*, which are induced by 10 mM Mn^2+^, was also effectively prevented by adding LaCl_3_ (data not shown).

Ca^2+^ participates in regulating cellulase production in *T. reesei* [13]. To investigate whether a cellulase increase, induced by Mn^2+^, was blocked by LaCl_3_, we analyzed CMCase and *p*NPCase activities and transcription of key cellulase genes *cbh1* and *egl1*. As shown in Fig. 5C, D (Additional file 8: Figure S9A, B), under Mn^2+^ supplementation and with LaCl_3_, the CMCase and *p*NPCase activities in *T. reesei* Rut-C30 decreased by 50 and 49%, respectively, compared with no LaCl_3_ addition. Meanwhile, the expression levels of *cbh1* and *egl1* were reduced by 60% at 72 h (Fig. 5E, F). However, there was no obvious change to CMCase activities and transcriptional levels of *cbh1* and *egl1* with or without LaCl_3_ when Mn^2+^ was not added (Fig. 5C–F, Additional file 8: Figure S9A, B). These results showed that LaCl_3_ could significantly decrease the Mn^2+^-induced high expression levels of key cellulase genes in *T. reesei* Rut-C30.

These data indicated that Mn^2+^ induced cytosolic Ca^2+^ increase via the Ca^2+^ channel. When a Ca^2+^ channel blocker LaCl_3_ was added, the increase in cytosolic Ca^2+^ concentration and cellulase production induced by Mn^2+^ were effectively attenuated.

**Fig. 6:**
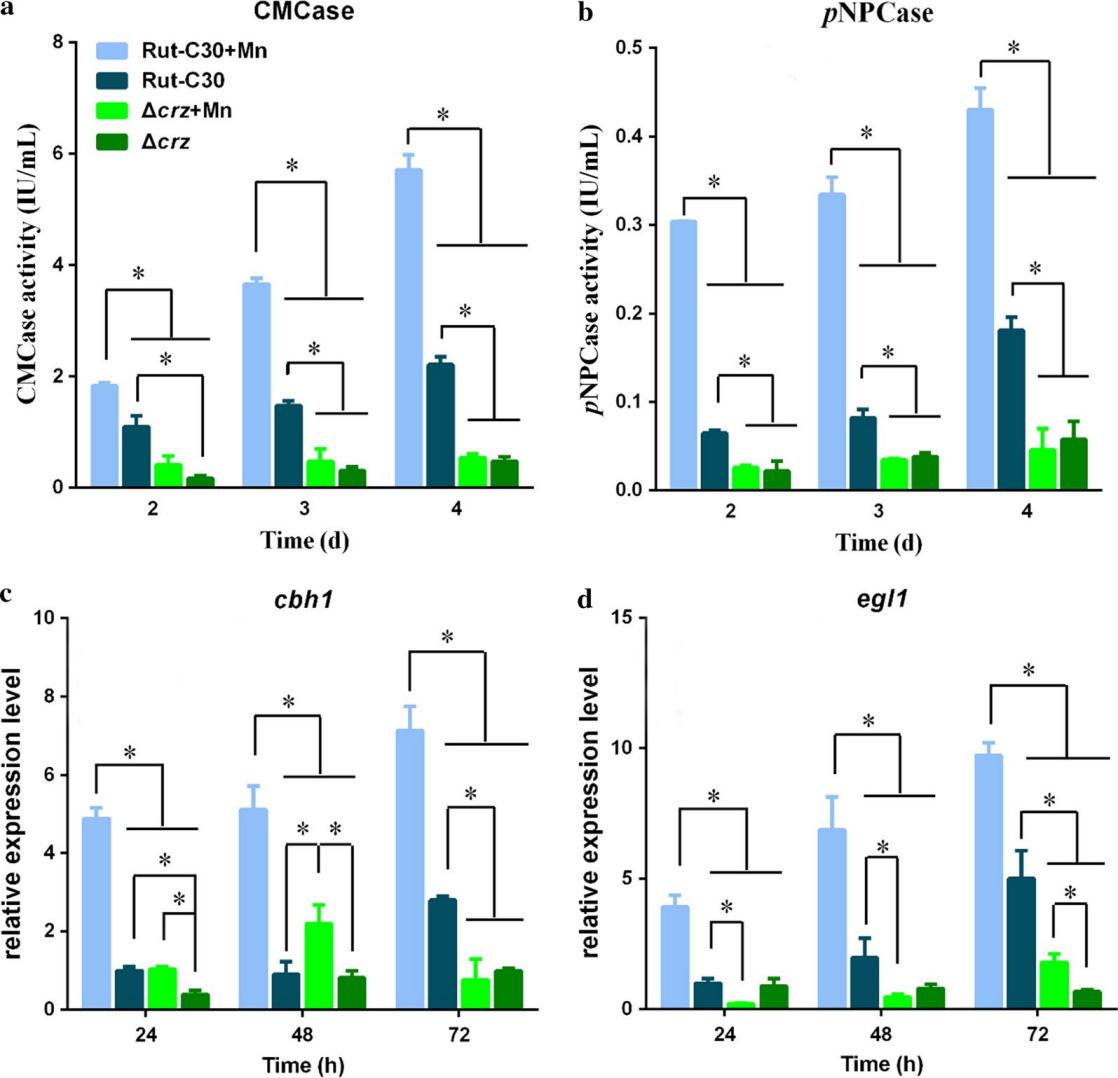
Influence of CRZ1 on Mn^2+^-induced cellulase production. The CMCase activity (**a**) and *p*NPCase activity (**b**) of *T. reesei* Rut-C30 and Δ*crz1* strains supplemented with 0 or 10 mM MnCl_2_. The expression levels of *cbh1* (**c**) and *egl1* (**d**) in *T. reesei* Rut-C30 and Δ*crz1* strains supplemented with 0 or 10 mM MnCl_2_. Values are the mean ± SD of the results from three independent experiments. Asterisks indicate significant differences (*p < 0.05, Student’s *t* test)

To investigate whether a cellulase increase, induced by Mn^2+^, is associated with calcium signal transduction, we constructed a *crz1* deletion mutant Δ*crz1* as Chen et al. [13] to block the calcium signal transduction pathway. As shown in Fig. 6a, b (Additional file 8: Figure S10A, B), the remarkable increase of CMCase and *p*NPCase activities induced by Mn^2+^, observed in parental strain Rut-C30, was effectively attenuated by deleting *crz1*. Similarly, the transcriptional levels of *cbh1* and *egl1* were markedly reduced in the Δ*crz1* mutant at all time points examined (Fig. 6c, d).

Taken together, these data indicated that Mn^2+^ induces cellulase improvement via calcium signal transduction in *T. reesei* Rut-C30. The improvement of cellulase production induced by Mn^2+^ is effectively prevented in a *crz1* mutant, blocking the calcium signal transduction pathway.

### TPMR1 functions in conjunction with channels of Mn^2+^ and Ca^2+^

Mn^2+^/Ca^2+^ homeostasis exists in other fungi [33–36]. PMR1 plays an important role in Mn^2+^/Ca^2+^ homeostasis, and may act as a Ca^2+^/Mn^2+^ exchanger to balance Mn^2+^ via Ca^2+^ [17]. To clarify how Mn^2+^ increased cytosolic Ca^2+^ concentration, using *Neurospora crassa* PMR1 (GenBank: CAB65296.1) as the query, we searched for a PMR1 homolog (TRE119592) in the *T. reesei* genome, named as TPMR1.

**Fig. 7:**
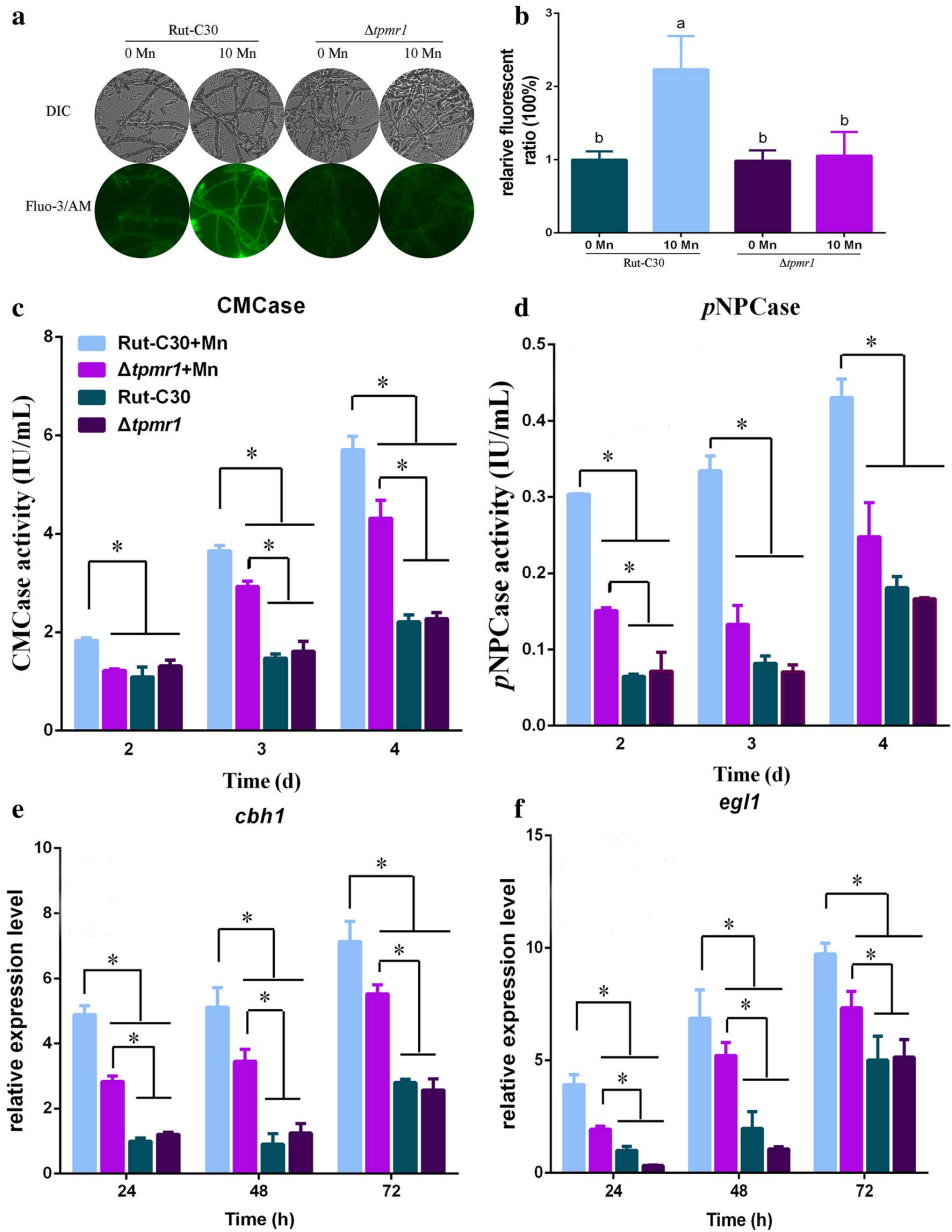
Influence of TPMR1 on Mn^2+^-induced cytosolic Ca^2+^ burst and cellulase production. **A** Fluorescence analysis of the influence of TPMR1 on the cytosolic Ca^2+^ burst induced by Mn^2+^. The *T. reesei* Rut-C30 and Δ*tpmr1* strains were cultured in liquid minimal medium for 48–60 h with 0 or 10 mM MnCl_2_ (0 or 10 Mn, respectively). For detection, 50 μM Fluo-3/AM was used, and the intensity was monitored using Automatic Inverted Fluorescence Microscopy. Green fluorescence represents the free cytosolic Ca^2+^. DIC, differential interference contrast. **B** Comparative fluorescence ratio analysis of TPMR1 influence on cytosolic Ca^2+^ burst induced by Mn^2+^. The *y*-axis represents the Ca^2+^ fluorescence ratio measured by CLSM and the *x*-axis the different treatments. The CMCase activity (**C**) and *p*NPCase activity (**D**) of *T. reesei* Rut-C30 and Δ*tpmr1* strains were examined after culture in medium containing 0 or 10 mM MnCl_2_. The expression levels of *cbh1* (**E**) and *egl1* (**F**) in *T. reesei* Rut-C30 and Δ*tpmr1* strains were analyzed after culture in medium containing 0 or 10 mM MnCl_2_. Values are the mean ± SD of the results from three independent experiments. Asterisks indicate significant differences from untreated strains (*p < 0.05, **p < 0.01, Student’s *t* test). Different letters indicate significant differences between the columns (*p* < 0.05, according to Duncan’s multiple-range test)

We constructed a *tpmr1* deletion mutant Δ*tpmr1*. To investigate whether TPMR1 is responsible for pumping in Ca^2+^ upon 10 mM Mn^2+^ addition in *T. reesei*, we measured the level of intracellular Ca^2+^ in the Δ*tpmr1* strain with or without Mn^2+^ addition. As shown in Fig. 7A and B, the increase in intracellular Ca^2+^ induced by Mn^2+^ in Rut-C30 was absent in the Δ*tpmr1* strain. The augmentation of Ca^2+^ levels induced by Mn^2+^ was blocked in the Δ*tpmr1* strain. These results suggested that TPMR1 is responsible for the increase of cytosolic Ca^2+^ under Mn^2+^ addition in *T. reesei* Rut-C30.

To further investigate the function of TPMR1 in conjunction with Mn^2+^ and Ca^2+^ channels, the intracellular and extracellular Mn^2+^ concentrations in the Δ*tpmr1* strain with or without Mn^2+^ addition were measured by ICP-MS during cultivation. As illustrated in Additional file 9: Figure S12, upon Mn^2+^ addition (10 mM final concentration), the intracellular Mn^2+^ concentration initially markedly increased, reaching and maintaining a high level after 24 h. The extracellular Mn^2+^ concentration dropped initially, reaching its minimum from 24 to 72 h. The intracellular and extracellular Mn^2+^ concentrations of the Δ*tpmr1* strain (Additional file 9: Figure S12) are quite different from that of Rut-C30 (Fig. 2a), whose intracellular Mn^2+^ gradually effused into the medium. These results suggested that TPMR1 is responsible for pumping Mn^2+^ out of the cell.

To further test the effect of TPMR1 on cellulase production under Mn^2+^ addition, we compared the cellulase production in parental strain Rut-C30 and Δ*tpmr1* mutant. Upon the addition of Mn^2+^, the Δ*tpmr1* strain caused about 26 and 51% reductions in the CMCase and *p*NPCase activities, respectively, compared with that of Rut-C30 (Fig. 7C, D and Additional file 8: Figure S11A, B). Moreover, the transcriptional levels of *cbh1* and *egl1* observed are in agreement with the CMCase and *p*NPCase activity levels, which were markedly reduced in the Δ*tpmr1* mutant at all time points examined (Fig. 7E, F). The increase in cellulase production induced by Mn^2+^ was remarkably weakened in the *tpmr1* deletion mutant. These results demonstrated that TPMR1 functions in conjunction with channels of Mn^2+^ and Ca^2+^ in *T. reesei* Rut-C30.

## Discussion

Metal ions are important in regulating cellular metabolism. For example, Ca^2+^ plays an important role in the regulation of cellulase or hemicellulase in *T. reesei* [13–15]. Paraszkiewicz et al. [37] reported Cd^2+^, Zn^2+^, and Pb^2+^ as environmental stress factors, which increased the biosynthesis of fungal emulsifier in *Curvularia lunata*. Addition of Ca^2+^, Na^+^, and Mn^2+^ enhanced ganoderic acid production in *Ganoderma lucidum* liquid cultures, through induction of the calcineurin signal pathway [17, 29, 38]. These results suggest that metal ions have significant impact on the cellular physiology and metabolism of various organisms. In this study, we first report that Mn^2+^ stimulates cellulase production and total protein secretion of *T. reesei* Rut-C30. However, whether other metal ions or stimuli have an impact on cellulase production of *T. reesei* will require further evidence for confirmation.

Diverse Mn^2+^ transports have been identified in the yeasts, plants, and bacteria [21, 22, 39–42]. Mn^2+^ can enter the cell through two different routes including high- and low-affinity transporters [21, 22, 41, 42]. SMF1, a plasma membrane NRAMP (Natural Resistance-Associated Macrophage Protein) family Mn^2+^ transporter, is a high-affinity transporter for Mn^2+^, contributing to Mn^2+^ accumulation under extreme Mn^2+^ starvation conditions [16, 22, 43, 44]. PHO84, a family of phosphate/proton symporters [45], can transport Mn^2+^ under excess Mn^2+^ stress [16, 25], indicating that PHO84 is a low-affinity transporter of Mn^2+^, contributing to Mn^2+^ accumulation only when cells are exposed to higher Mn^2+^ concentrations. *T. reesei* might contain both high- and low-affinity transporters of Mn^2+^, similar to what has been described for the metal transport systems for iron, copper, and zinc [46–50]. A BLAST search of the genome sequence of *T. reesei* identified a gene (*tre79644*) similar to SMF1, and this gene was named *tsmf1*. There was no significant difference on Mn^2+^ transport and cellulase production under 10 mM Mn^2+^ surplus using a *tsmf1* deletion strain (data not shown). The alternative PHO84 homologs remain to be identified in *T. reesei* Rut-C30. Five candidates including TPHO84-1 and TPHO84-2 are similar to PHO84. We proved that extracellular Mn^2+^ is transported into Rut-C30 cells via TPHO84-1 and TPHO84-2, which are annotated as putative low-affinity transports of Mn^2+^ responding to higher Mn^2+^ concentration. Meanwhile, there were no significant differences between the cytosolic Ca^2+^ concentrations for the *T. reesei* Rut-C30, Δ*tpho84*-*1*, Δ*tpho84*-*2*, and Δ*tpho84*-*1/2* mutants (see Additional file 10: Figure S13).

Calcium is widely used as a second messenger in prokaryotic and eukaryotic cells. It is known that a cytosolic Ca^2+^ burst and further induced calcium signaling regulate cellular responses when exposed to different external stimuli [51, 52]. For instance, Na^+^ induction enhances cytosolic Ca^2+^ to induce the ganoderic acid biosynthesis through calcineurin signal pathway, to upregulate its biosynthetic genes at the transcriptional level in *Ganoderma lucidum* [29]. High temperatures are known to trigger the generation of cytosolic Ca^2+^ in plants [53]. Alkaline pH triggers an immediate calcium burst in *Candida albicans* [54]. Our results indicated that Mn^2+^ induces a significantly increased cytosolic Ca^2+^ level in *T. reesei* Rut-C30 (Fig. 4A, B). Additionally, in our experiments, inhibition of cytosolic Ca^2+^ level by LaCl_3_ effectively attenuated the cellulase increase induced by Mn^2+^ (Fig. 5C–E). The results showed that 10 mM Mn^2+^ stress led to a cytosolic Ca^2+^ burst in *T. reesei* Rut-C30. An earlier study demonstrated that Ca^2+^ (/calmodulin)–calcineurin–CRZ1 signaling could induce cellulase production at the transcription level by Ca^2+^ stimulation [13]. In our study, Mn^2+^ could also activate the expression of Ca^2+^ (/calmodulin)–calcineurin–CRZ1 signaling-related genes in *T. reesei* Rut-C30 (Fig. 4C–E). Furthermore, CRZ1 participated in regulating cellulase production in Mn^2+^-induced strains (Fig. 6a–d). The results presented here indicate that the regulation of cellulase gene expression and production by Mn^2+^ are dependent on cytosolic Ca^2+^ burst and further induce calcium signaling.

PMR1, encoding the Ca^2+^/Mn^2+^ P-type ATPase, is required to either scavenge trace amounts of Mn^2+^ and Ca^2+^ from the medium or maintain sufficient levels of Mn^2+^ and Ca^2+^ in an intracellular compartment [22, 24–26, 33]. Bowman et al. [33] reported that a *pmr1* deletion strain accumulates 80% lesser Ca^2+^ than the wild type. Under higher levels of intracellular Mn^2+^, extracellular Ca^2+^ might transport into the cells through PMR1 to increase intracellular Ca^2+^ and then trigger calcium signaling to regulate cellular responses [35]. Our work found that the increase in Ca^2+^ levels induced by Mn^2+^ treatment was blocked in the *tpmr1* deletion strain (Fig. 7A, B) and that the improvement of cellulase production induced by Mn^2+^ was remarkably weakened in the *tpmr1* deletion mutant. These results demonstrated that TPMR1 functions as a channel of Mn^2+^ and Ca^2+^ in *T. reesei* Rut-C30. Cytosolic Ca^2+^ concentration was enhanced through TPMR1 under Mn^2+^ addition, thus inducing calcium signaling to upregulate cellulase genes. Additionally, PMR1 provides a major route for cellular sequestration of Mn^2+^ by pumping excess Mn^2+^ into the Golgi, from where the metal may exit the cell via the secretory pathway vesicles that merge with the cell surface and release the Mn^2+^ contents back into the extracellular environment [20, 22, 24, 34]. These data correlate well with our data indicating that intracellular Mn^2+^ gradually effused to the medium from 24 h (Fig. 2a). However, in the Δ*tpmr1* mutant, the intracellular Mn^2+^ concentration maintained its high level after 24 h, and the extracellular Mn^2+^ concentration reached its minimum from 24 to 72 h. These results implied that TPMR1 participates in pumping excess Mn^2+^ into the Golgi, and then releases it extracellularly, meanwhile accumulating cytosolic Ca^2+^ in *T. reesei* Rut-C30. However, the detailed role of TPMR1 in *T. reesei* needs further research.

We found that 10 mM Mn^2+^ could also stimulate cellulase production and increase total protein secretion from the *T. reesei* wild-type strain QM6a and the mutant strain Qm9414 (data not shown). However, the results from Rut-C30 may not be identical to those of the wild-type strain, because each strain likely has its own unique regulatory mechanism.

## Conclusions

**Fig. 8:**
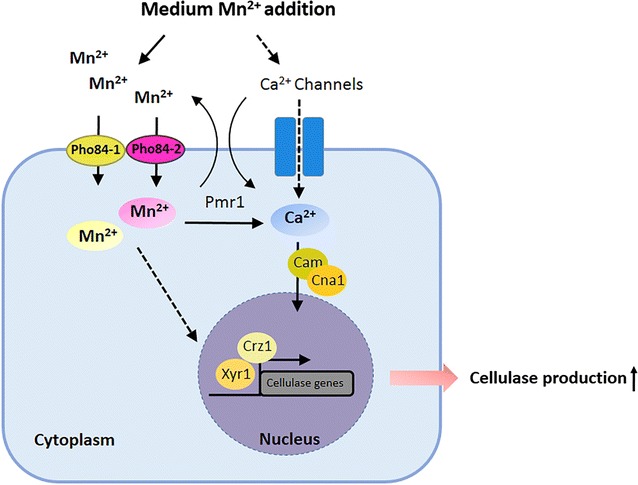
A mechanistic model of the Mn^2+^ stimulation of cellulase production in *T. reesei*. After addition of 10 mM MnCl_2_, the intracellular Mn^2+^ content increases via Mn^2+^ transport proteins Tpho84-1 and Tpho84-2. Intracellular Mn^2+^ promotes a cytosolic Ca^2+^ burst that is required for cellulase gene transcription via Ca^2+^ signaling. After using LaCl_3_ (plasma membrane Ca^2+^ channels blocker), we suggest that Ca^2+^ channels are responsible for the cytosolic Ca^2+^ burst and cellulase production induced by Mn^2+^. Furthermore, TPMR1 is one of the links between the channels of Mn^2+^ and Ca^2+^, which may function as a Mn^2+^/Ca^2+^ exchanger to regulate Mn^2+^ and Ca^2+^ homeostasis under Mn^2+^ stress. Mn^2+^ could also employ other as-yet-unidentified pathways to regulate cellulase production. The solid arrows indicate data supported by our own experiments; dashed arrows indicate undefined regulation

In summary, the putative mechanism of the extracellular Mn^2+^-induced stimulation of cellulase production was characterized in *T. reesei* Rut-C30 (Fig. 8). Mn^2+^ induces a significantly increased cytosolic Ca^2+^ level and triggers Ca^2+^-CRZ1 signaling to induce cellulase production at the transcription level. Moreover, we identified two Mn^2+^ transport proteins in *T. reesei* Rut-C30, and named TPHO84-1 and TPHO84-2. Furthermore, TPMR1 acts as a link between channels in Mn^2+^ and Ca^2+^ homeostasis in *T. reesei*. This study provides a successful approach to produce a higher yield of cellulase and to develop industrially applicable *T. reesei* strains, which is important for biofuel production from lignocelluloics. This study also provides a molecular basis for understanding the regulatory mechanism of divalent metal ions on the cellular metabolism of fungi.

## Methods

### Strains and growth conditions

*Escherichia coli* DH5α was used for plasmid amplification. *Agrobacterium tumefaciens* strain AGL-1 was used as a T-DNA donor for fungal transformation [55]. *T. reesei* Rut-C30 (ATCC 56765) was used throughout the study and as the host for genetic transformation. Luria–Broth (LB) was used for culture of *E. coli* and *A. tumefaciens*. Mandels’ medium [56] was used for the general fungal culture. All strains were maintained on potato dextrose agar (PDA) plates at 28 °C. The fungal strains constructed in this study are summarized in Table 1. All strains were cultured in the dark.

**Table 1:** *T. reesei* strains constructed in this study

Strains	Relevant features	Source
Rut-C30	Parent strain	ATCC
Δ*tpho84*-*1*(Δ*77552*)	Rut-C30-deleted *tpho84*-*1* gene	This study
Δ*tpho84*-*2*(Δ*45868*)	Rut-C30-deleted *tpho84*-*2* gene	This study
Δ*tpho84*-*1/2*	Rut-C30-deleted *tpho84*-*1* and *tpho84*-*2* gene	This study
Δ*81389*	Rut-C30-deleted *tre81389* gene	This study
Δ*45852*	Rut-C30-deleted *tre45852* gene	This study
Δ*106118*	Rut-C30-deleted *tre106118* gene	This study
rfp-*tpho84*-*1*	Rut-C30-harboring vector prfp-*tpho84*-*1*	This study
rfp-*tpho84*-*2*	Rut-C30-harboring vector prfp-*tpho84*-*2*	This study
*tpho84*-*1*-rc	Rut-C30-harboring vector p*tpho84*-*1*-rc	This study
*tpho84*-*2*-rc	Rut-C30-harboring vector p*tpho84*-*2*-rc	This study
Δ*crz1*	Rut-C30-deleted *crz1* gene	This study
Δ*tpmr1*	Rut-C30-deleted *tpmr1* gene	This study

Minimal medium (MM, (NH_4_)_2_SO_4_ 5 g/l; Urea 0.3 g/l; KH_2_PO_4_ 15 g/l; CaCl_2_ 0.6 g/l; MgSO_4_ 0.6 g/l; FeSO_4_·7H_2_O 5 mg/l; ZnSO_4_·7H_2_O 1.4 mg/l; CoCl_2_·6H_2_O 2 mg/l; pH 5.5) with 2% glucose was used to assess the effect of Mn^2+^ on hyphal growth. To analyze the effects of Mn^2+^ on cellulase activity, protein concentration, and gene expression levels, medium replacement experiments were performed. After growth in Mandels’ medium with 2% glucose for ~ 32 h at 28 °C and 220 rpm, approximately 0.1 g of mycelia were collected and washed thoroughly using 0.85% NaCl, then transferred to 100 ml MM containing 1% (w/v) Avicel (PH-101, Sigma-Aldrich) with the addition of MnCl_2_ to final concentrations of 1, 10, 20, and 40 mM. Strains were induced for 1–5 days before being subjected to testing for enzymatic activity, protein concentration, or induced for 24, 48, or 72 h before being subjected to RNA extraction and RT-qPCR analyses, respectively. To assess the effect of plasma membrane Ca^2+^ channels on the regulation of cellulase production of *T. reesei* Rut-C30, 5 mM (final concentration) LaCl_3_ (Aladdin, Shanghai, China) was added after 1 day of culture in MM.

### Fungal growth, enzymatic activity, protein concentration, and biomass assays

For fungal hyphal growth assays, conidia were collected and diluted to 10^7^ ml^−1^ in sterile water. An equal volume of the solution (2 μl) was inoculated onto the center of the MM plates as described above, and was grown for 3–5 days at 28 °C.

For enzymatic activity, protein concentration, and biomass assays, 1 ml of culture liquid was collected and subjected to 0.45-μm filtration. The culture supernatants were subjected to cellulase activity and protein concentration analysis. The mycelia were subjected to biomass measurement. Fungal CMCase and *p*NPCase activities were measured according to the method described by Wang et al. [57]. Protein concentrations were determined using the Bradford Protein Assay Kit (Generay, Shanghai, China). Biomass concentration was indirectly measured by calculating the amount of total intracellular proteins, with some modification [58]. Briefly, harvested mycelia were suspended in 1 ml 1 M NaOH in a reaction tube and the mixture was incubated for 2 h and frequently vortexed. Total protein was collected via centrifugation at 14,000×*g* at 4 °C for 10 min. Total protein concentration was determined by the Modified Lowry Protein Assay Kit (Sangon Biotech, Shanghai, China). The final protein content was furthermore corrected using a set of substrate controls where no inoculum was added to the medium. The biomass dry weight was then calculated assuming an average content of 0.32 g intracellular protein per gram of dry cell mass.

### RNA isolation and quantitative real-time reverse-transcription polymerase chain reaction (RT-qPCR)

The levels of gene-specific mRNA were assessed using RT-qPCR, according to our previous study, with some modification [59]. In brief, the total RNA of 50 mg fresh weight cells was extracted using a FastRNA Pro Red Kit (MPbio, Irvine, CA, USA), according to the manufacturer’s instructions. Synthesis of cDNA from total RNA was performed using the PrimeScript RT Reagent Kit with gDNA eraser (TaKaRa, Japan) as per the manufacturer’s instructions. For RT-qPCR, the TransStart TipTop Green qPCR SuperMix (TransGen, Shanghai, China) was used with 200 nM of forward and reverse primers (see Additional file 1: Table S1). Gene transcription was analyzed using SYBR green assays. Transcription levels of target genes were normalized to that of the *sar1* gene [60]. Thermocycling was performed in an ABI StepOne Plus thermocycler (Applied Biosystems, Foster City, CA, USA).

### Determination of extracellular and intracellular Mn^2+^ concentration

Extracellular and intracellular Mn^2+^ concentrations were measured by inductively coupled plasma mass spectrometry (ICP-MS), as described for *Ganoderma lucidum* by Xu [17]. Five milliliter of culture liquid was collected and subjected to filtration. The culture supernatant and mycelium were subjected to extracellular and intracellular Mn^2+^ concentrations, respectively. The supernatant of the cultures was filtered through 0.22-μm membranes and then diluted with 1% HNO_3_ for measuring the extracellular Mn^2+^ concentration. Mycelia were washed in distilled water to remove any nonspecifically bound Mn^2+^, and digested with 1 ml 68% HNO_3_. The mixture was collected via centrifugation at 12,000×*g* for 5 min. The supernatant was then filtered through a 0.22-μm membrane and diluted with 1% HNO_3_ for measuring intracellular Mn^2+^ concentration. The final intracellular Mn^2+^ concentration was shown as micromole per gram biomass.

### Construction of plasmids and strains

To construct a *tpho84*-*1* deletion mutant, the upstream (− 1 to − 762 bp) and downstream (+ 1917 to + 2681 bp) fragments of *tpho84*-*1* were generated from the genome of *T. reesei* Rut-C30 using KOD-Plus-Neo (TOYOBO, Japan). All primers are indicated in Additional file 1: Table S1. First, the upstream fragment was ligated into the *Pac*I- and *Xba*I-linearized LML2.0 [61] using the ClonExpressTM II One Step Cloning Kit (Vazyme, Nanjing, China) to form pF*tpho84*-*1*. Subsequently, the downstream fragment was inserted into *Swa*I-linearized pF*tpho84*-*1* to form the binary vector pD*tpho84*-*1* (see Additional file 5: Figure S4A) for the knockout of *tpho84*-*1* in Rut-C30 using *Agrobacterium*-mediated transformation [59]. Strains were selected using hygromycin B and cefotaxime on Mandels’ medium. Then, the marker was excised using the method described by Zhang [61]. The putative *tpho84*-*1* disruption mutants (Δ*tpho84*-*1*/Δ*77552*) generated by double crossover were verified by diagnostic PCR using the primers tpho84-1-CF and tpho84-1-CR and tpho84-1-OF and tpho84-1-OR (see Additional file 5: Figure S4E).

Similarly, the 706-bp upstream and 710-bp downstream regions of *tpho84*-*2* were amplified and then inserted into LML2.0 as described above to generate the binary vector pD*tpho84*-*2* (see Additional file 5: Figure S4B). After transformation and selection, the putative *tpho84*-*2* deletion mutant (Δ*tpho84*-*2*/Δ*45868*) was verified by diagnostic PCR using the primers tpho84-2-CF and tpho84-2-CR, and tpho84-2-OF and tpho84-2-OR (see Additional file 5: Figure S4E). The *tre81389*, *tre45852*, *tre106118*, *crz1*, and *tpmr1* disruption mutants (Δ*81389*, Δ*45852*, Δ*106118*, Δ*crz1*, and Δ*tpmr1*) were constructed similarly (see Additional file 11: Figure S14).

To construct a *tpho84*-*1/2* deletion mutant, the pD*tpho84*-*2* cassette was transformed into Δ*tpho84*-*1* strain using *Agrobacterium*-mediated transformation. The *tpho84*-*1/2* double deletion mutant (Δ*tpho84*-*1/2*) was verified by diagnostic PCR.

For *tpho84*-*1* and *tpho84*-*2* re-complementation, 3916- and 4087-bp DNA fragments containing total *tpho84*-*1* and *tpho84*-*2* expression cassette were amplified from the Rut-C30 genome using tpho84-1-rc-1/tpho84-1-rc-2 and tpho84-2-rc-1/tpho84-2-rc-2, and then inserted into *Swa*I-linearized LML2.0 to generate vector p*tpho84*-*1*-rc and p*tpho84*-*2*-rc, respectively. P*tpho84*-*1*-rc and p*tpho84*-*2*-rc were then transferred to the *tpho84*-*1* and *tpho84*-*2* deletion strain by *Agrobacterium*-mediated transformation (see Additional file 5: Figure S4C, D). *tpho84*-*1* and *tpho84*-*2* re-complementation strains (*tpho84*-*1*-rc and *tpho84*-*2*-rc) were selected and verified by PCR using tpho84-1-OF and tpho84-1-OR, and tpho84-2-OF and tpho84-2-OR (see Additional file 5: Figure S4F).

For the construction of N-terminal RFP-tagged translational fusion of *tpho84*-*1* under the control of the *cbh1* promoter, the upstream (− 1 to − 1038 bp) and downstream (+ 1 to + 1084 bp) fragments of *tpho84*-*1* were generated from the genome of *T. reesei* Rut-C30 using KOD-Plus-Neo (TOYOBO, Japan). The promoter of *cbh1* (P_*cbh1*_) was obtained by PCR from the genome of *T. reesei* Rut-C30. Red fluorescent protein (*rfp*) was obtained by PCR using the plasmid pDsRed2-N1 (Clontech) as the template. First, the upstream fragment was ligated into the *Pac*I- and *Xba*I-linearized LML2.0, using the ClonExpressTM II One Step Cloning Kit (Vazyme, Nanjing, China) to form pFr*tpho84*-*1*. Subsequently, the P_*cbh1*_, *rfp*, and downstream fragments were inserted into *Swa*I-linearized Fr*tpho84*-*1* to form the binary vector pRFP-TPHO84-1 for subcellular location of *tpho84*-*1* using *Agrobacterium*-mediated transformation. Subsequently, the marker was excised following the method of Zhang [61]. The putative rfp-*tpho84*-*1* mutants (rfp-*tpho84*-*1*) generated by double crossover were verified by diagnostic PCR using the primers rfp-tpho84-1-CF and rfp-tpho84-1-CR (see Additional file 12: Figure S15).

Similarly, the 997-bp upstream and 797-bp downstream regions of *tpho84*-*2* were amplified and then inserted into LML2.0 as described above to generate the binary vector pRFP-TPHO84-2 (see Additional file 6: Figure S5A). After transformation and selection, the putative rfp-*tpho84*-*2* mutants (rfp-*tpho84*-*2*) was verified by diagnostic PCR using the primers rfp-tpho84-2-CF and rfp-tpho84-2-CR (see Additional file 12: Figure S15).

The genome sequence of *T. reesei* is available at the US Department of Energy (DOE) Joint Genome Institute (http://genome.jgi-psf.org/Trire2/Trire2.home.html).

### Fluorescence microscopy

To localize RFP-TPHO84-1/2 fusion proteins using microscopy, the rfp-*tpho84*-*1* and rfp-*tpho84*-*2* strains were inoculated into Mandels’ medium and grown for 48–60 h. The mycelia were then observed using an S Plan Fluor ELWD 100×, 1.3 numerical aperture (NA) objective on a Laser Scanning Confocal Microscope (A1R, Nikon, Japan) comprising a Texas Red filter (500–620 nm band-pass excitation filter and emission filter of 670 nm). Images were processed using the NIS elements software (Nikon).

### Free cytosolic Ca^2+^ labeling and detection

Fluo-3/AM (Sigma) was used as a Ca^2+^-specific probe to assess the level of cytoplasmic Ca^2+^ in *T. reesei* Rut-C30 according to the manufacturer’s protocol. Fluo-3/AM (50 μM final concentration) was loaded into cells by incubation at 37 °C for 30 min, and the cells were then washed three times with phosphate-buffered saline. Images of Ca^2+^ green fluorescence were observed using an S Plan Fluor ELWD 20×, 0.5 numerical aperture (NA) objective and a digital sight camera on an Eclipse Ti inverted microscope system (Ti-E, Nikon, Japan), comprising an FITC filter (420–490 nm band-pass excitation filter, and emission filter of 535 nm). The intensity of green fluorescence was quantified using NIS-Elements F package software. To eliminate the contribution of background fluorescence, cells without Fluo-3/AM labeling were also imaged under identical conditions.

### Statistical analysis

All experimental data shown in this paper were carried out at least three times with identical or similar results. For every experiment, three biological replicates were performed with three technical replicates. The error bars indicate the standard deviation (SD) from the mean of triplicates. Student’s *t* test was used to compare two samples. Duncan’s multiple-range test was used for multiple comparisons. *p* < 0.05 was considered to be significant.

## Additional files

**Additional file 1: Table S1.** Primers used in this study.

**Additional file 2: Figure S1.** Influence of Mn^2+^ on the transcriptional levels of cellulase-encoding genes in *T. reesei* Rut-C30. **A**–**E** The effects of Mn^2+^ on the transcriptional levels of *cbh1* (A), *cbh2* (B), *xyr1* (C), *egl1* (D) and *egl2* (E). 0 Mn, no Mn^2+^ was added to the medium; 10 Mn, final concentration of 10 mM. Three independent experiments with three biological replicates each were performed. Values are the means ± SD of the results from three independent experiments. Asterisks indicate significant differences from untreated strains (*p< 0.05, **p < 0.01, Student’s *t* test).

**Additional file 3: Figure S2.** Influence of Mn^2+^ addition on the transcriptional levels of putative Mn^2+^ transport-encoding genes. **A**–**E** The effect of Mn^2+^ on the transcriptional levels of *tre77552* (A), *tre81389* (B), *tre45852* (C), *tre45868* (D), and *tre106118* (E). 0 Mn, no Mn^2+^ was added to the medium; 10 Mn, final concentration of 10 mM. Values are the means ± SD of the results from three independent experiments. Asterisks indicate significant differences from untreated strains (*p< 0.05, Student’s *t* test).

**Additional file 4: Figure S3.** Concentrations of Mn^2+^ in *T. reesei* Rut-C30 and its derivative mutants. The concentrations of intracellular Mn^2+^ of *T. reesei* Rut-C30 and its derivative mutant strains were examined after cultured in medium containing 10 mM MnCl_2_. Values are the means ± SD of the results from three independent experiments. Asterisks indicate significant differences from parental strain Rut-C30 (*p< 0.05, Student’s *t* test).

**Additional file 5: Figure S4.** Construction and verification of Δ*tpho84-1*, Δ*tpho84-2*, *tpho84-1*-rc, *tpho84-2*-rc mutants. (A) Schematic representation of the *tpho84-1* locus from Rut-C30 and Δ*tpho84-1* mutant. The binding sites of primers on the genome of Rut-C30 and Δ*tpho84-1*, and the expected sizes of the products in PCR verification are given. The region from +1 to +1916 bp relative to the translation start site of *tpho84-1* (grey box) was replaced with the hygromycin resistance expression cassette (hygromycin, black box). (B) Schematic representation of the *tpho84-2* locus from Rut-C30 and Δ*tpho84-2* mutant. The binding sites of primers on the genome of Rut-C30 and Δ*tpho84-2* and the expected sizes of the PCR products are given. The region from +1 to +2087 bp relative to the translation start site of *tpho84-2* (grey box) was replaced with the hygromycin resistance expression cassette (hygromycin, black box). (C) Schematic representation of the P-tpho84-1-tpho84-1-T-tpho84-1 cassette in *tpho84-1*-rc strains. The primer pairs indicated were used in the verification of the expression cassette. P-tpho84-1, the possible promoter of *tpho84-1*; T-tpho84-1, the possible terminator of *tpho84-1*. (D) Schematic representation of the P-tpho84-2-tpho84-2-T-tpho84-2 cassette in *tpho84-2*-rc strains. The primer pairs indicated were used in the verification of the expression cassette. P-tpho84-2, the possible promoter of *tpho84-2*; T-tpho84-2, the possible terminator of *tpho84-2*. (E) PCR verification of the Δ*tpho84-1* and Δ*tpho84-2* mutants. Lane M, DNA molecular mass maker. PCR amplification results of the Dtpho84-1-F were obtained using tpho84-1-CF with D70-4 and Dtpho84-1-R were obtained using HG3.6 with tpho84-1-CR (see Additional file 1: Table S1). PCR amplification results of the Dtpho84-2-F were obtained using tpho84-2-CF with D70-4 and Dtpho84-2-R were obtained using HG3.6 with tpho84-2-CR. (F) PCR verification of the *tpho84-1*-rc and *tpho84-2*-rc strains. Lane M, DNA molecular mass maker. PCR amplification results of the *tpho84-1*-rc were obtained using tpho84-1-OF with tpho84-1-OR and *tpho84-2*-rc was obtained using tpho84-2-OF with tpho84-2-OR. The genome of the Δ*tpho84-1* and Δ*tpho84-2* mutants were used as a negative control.

**Additional file 6: Figure S5.** Subcellular localization of TPHO84-1 and TPHO84-2. Schematic representation of the plasmids used for expression of RFP-TPHO84-1 and RFP-TPHO84-2. RFP was fused to the N-terminal of TPHO84-1 and TPHO84-2 and expressed under the control of the *cbh1* promoter. **B.** In vivo epifluorescence analysis of rfp-*tpho84-1* and rfp-*tpho84-2* transformants. The transformants were cultured in liquid Mandels’ medium for 48–60 h and observed using Laser Scanning Confocal Microscopy.

**Additional file 7: Figure S6.** Cellulase activities of *T. reesei* Rut-C30 and its derivative mutant strains. The activities were examined after cultured in medium containing 0 mM Mn^2+^. (A) CMCase activities, (B) *p*NPCase activities. Values are the means ± SD of the results from three independent experiments.

**Additional file 8.** Data for corresponding cellulase activity per biomass. The CMCase/biomass activity and *p*NPCase/biomass activity of *T. reesei* strains were examined. For every experiment, three biological replicates were performed with three technical replicates each. Values are the means ± SD of the results from three independent experiments. Asterisks indicate significant differences (*p< 0.05, Student’s *t* test).

**Additional file 9: Figure S12.** Concentrations of Mn^2+^ in Δ*tpmr1* strain. The concentrations of extracellular and intracellular Mn^2+^ of the Δ*tpmr1* strain was examined after culturing in minimal medium containing 0 or 10 mM MnCl_2_. Filled circle, intracellular Mn^2+^ concentration after adding 10 mM MnCl_2_; blank circle, extracellular Mn^2+^ concentration after adding 10 mM MnCl_2_; filled square, intracellular Mn^2+^ concentration of control without MnCl_2_ supplementation; blank square, extracellular Mn^2+^ concentration of control without MnCl_2_ supplementation. Values are the means ± SD of the results from three independent experiments.

**Additional file 10: Figure S13.** Cytosolic Ca^2+^ levels increase after Ca^2+^ addition. **A.** The analysis of cytosolic Ca^2+^ levels via a Ca^2+^ fluorescent probe Fluo-3/AM. The *T. reesei* Rut-C30 and its derivative mutant strains were cultured in liquid minimal medium (initial 5.4 mM Ca^2+^) for 48–60 h with extra 0 or 10 mM CaCl_2_ supplementation (low Ca or high Ca, respectively). For detection, 50 μM Fluo-3/AM was used, and the intensity was monitored using Automatic Inverted Fluorescence Microscopy. Green fluorescence represents the free cytosolic Ca^2+^. DIC, differential interference contrast. **B.** Comparative fluorescence ratio analysis of Ca^2+^ influence on cytosolic Ca^2+^ levels. The *y*-axis represents the Ca^2+^ fluorescence ratio measured by CLSM, and the *x*-axis represents the different strains tested.

**Additional file 11: Figure S14.** Verification of Δ*81389*, Δ*45852*, Δ*106118*, Δ*crz1*, and Δ*tpmr1* mutants. Lane M, DNA molecular mass maker. PCR amplification results of the F were obtained using t81389-, t45852-, t106118-, tcrz1-, and ttpmr1-CF, respectively, with D70-4, and R were obtained using HG3.6 with t81389-, t45852-, t106118-, tcrz1-, and tpmr1-CR, respectively. PCR amplification using primer pairs t81389-, t45852-, t106118-, tcrz1-, and tpmr1-OF/OR, respectively, was performed as a negative confirm for gene deletion.

**Additional file 12: Figure S15.** PCR Verification of rfp-*tpho84-1*, rfp-*tpho84-2* mutants. Lane M, DNA molecular mass marker. PCR amplification results of the rfp-tpho84-1-F were obtained using rfp-tpho84-1-CF with D70-4 and rfp-tpho84-1-R were obtained using HG3.6 with rfp-tpho84-1-CR (Additional file 1: Table S1). PCR amplification results of the rfp-tpho84-2-F were obtained using rfp-tpho84-2-CF with D70-4 and rfp-tpho84-2-R were obtained using HG3.6 ith rfp-tpho84-2-CR.

## Abbreviations

*crz1*: calcineurin-responsive zinc finger transcription factor 1
CMCase: *endo*-β-glucanase activity
*p*NPCase: *exo*-β-glucanase activity
qRT-PCR: quantitative reverse-transcription PCR
ICP-MS: inductively coupled plasma mass spectrometry
NRAMP: Natural Resistance-Associated Macrophage Protein
