# Supplementary material for: Mn2+ modulates the expression of cellulase genes in Trichoderma reesei Rut-C30 via calcium signaling
Source: Biotechnol Biofuels. 2018 Mar 1;11:54. doi: 10.1186/s13068-018-1055-6 (PMC5831609; doi:10.1186/s13068-018-1055-6)
Supplement: Supplementary file 1 — Additional file 1: Table S1. Primers used in this study. [file 13068_2018_1055_MOESM1_ESM.docx]

**Table S1 Primers used in this study.**

| Primer | oligos Sequences (5’ to 3’) |
| --- | --- |
| **Quantitative RT-PCR analysis** | |
| Qsar1-1 | TGGATCGTCAACTGGTTCTACGA |
| Qsar1-2 | GCATGTGTAGCAACGTGGTCTTT |
| Qcbh1-1 | CTCCATCTCCGAGGCTCTTACC |
| Qcbh1-2 | GCAAGTGCCGCCATATCTGTTAT |
| Qcbh2-1 | GCATATTACGCCTCTGAAGTTAGCA |
| Qcbh2-2 | GCATAGTTACCGCCATTCTTGTTG |
| Qegl1-1 | GCAGCCTCACCATGAACCAGTA |
| Qegl1-2 | CACCGTCAGAGTCCAGGAGATAC |
| Qegl2-1 | TGAACAAGTCCGTGGCTCCAT |
| Qegl2-2 | ACAATTCGTAGGTCCGCTCCAA |
| Qxyr1-1 | CTTCCTCCTCCTGCTCATCG |
| Qxyr1-2 | TCGTGTGCCCTAACAATGGTC |
| QTr77552-1 | CCAGCTCGCCGTCGACAAGA |
| QTr77552-2 | TCGCGCTTGCCGGTCATGTA |
| QTr81389-1 | GTCGCCGGAGCCGGATTCTT |
| QTr81389-2 | GCGCTTGGTTGACGGTGTCG |
| QTr45852-1 | CGTCGAAGCCGACGCCCATA |
| QTr45852-2 | GGGATTCCCGCCGTTACCCA |
| QTr45868-1 | AGCGCCGTGTCTTCAGCCAT |
| QTr45868-2 | GCAGGTCGGCCCAGTAGGTG |
| QTr106118-1 | CGCTGGCCAGGAGCATCTGT |
| QTr106118-2 | ATTGTGCCGGATGGCGCTCT |
| **Construction of *tpho84-1* gene deletion vector** | |
| tpho84-1-D1 | ATTACGAATTCTTAATTAAGGCAGGATTCCGCAGACA |
| tpho84-1-D2 | CATTATACGAAGTTATTCTAGACACATTAGTGGCTCGCAGTC |
| tpho84-1-D3 | ACTAGTGAGCTCATTTCACGCGCTACAGTCTACAACAG |
| tpho84-1-D4 | AGTGCCAAGCTTATTTGCTTGCCGAACTCATCCTTGT |
| **Verification of the *tpho84-1* gene deletion mutants** | |
| tpho84-1-CF | CGCAGCGTGAAATCAGACAAA |
| tpho84-1-CR | AGGTAACCCGTAATGCGACATC |
| tpho84-1-OF | GCCTGCCTGCGATGTCATTG |
| tpho84-1-OR | TGAGAATGGTGCCGTTGTTCAG |
| D70-4 | TCGGACTTGCGGAGGATGTTGTAT |
| HG3.6 | TGCCTAGTGAATGCTCCGTAACA |
| **Construction of *tpho84-2* gene deletion vector** | |
| tpho84-2-D1 | ATTACGAATTCTTAATTAACAACGGCCTAACAGACGGATT |
| tpho84-2-D2 | CATTATACGAAGTTATTCTAGATGGACAGCAAGCGTGGATGA |
| tpho84-2-D3 | ACTAGTGAGCTCATTTAGGCGAAGCGTGGATAGAATGA |
| tpho84-2-D4 | AGTGCCAAGCTTATTTGTGGCAAGGAGGACAAGTCAGT |
| **Verification of the *tpho84-2* gene deletion mutants** | |
| tpho84-2-CF | GAGAGGCGAGGGCCATTCAT |
| tpho84-2-CR | GCAACAAAGTCATCACTCCTCC |
| tpho84-2-OF | TTGGAGCAGCAGCAGGAACA |
| tpho84-2-OR | AGGAAGGAGGCGGCGTAGTA |
| **Construction of *Tr81389* gene deletion vector** | |
| t81389-D1 | ATTACGAATTCTTAATTAACCATCCATCCATCCACCATCCA |
| t81389-D2 | CATTATACGAAGTTATTCTAGACGAGCGACTGAGCTGTGT |
| t81389-D3 | ACTAGTGAGCTCATTTCCTTTCCTCTTCCTCCCATCT |
| t81389-D4 | AGTGCCAAGCTTATTTACCGTTGTTGCACCATTGTTG |
| **Verification of the *Tr81389* gene deletion mutants** | |
| t81389-CF | AATACGACAAAGGCACGACTCG |
| t81389-CR | TGTCCGTATAGCATCGCTTTCA |
| t81389-OF | TTCTGGCGTGTCCTGATGGTAT |
| t81389-OR | GGCACTGAGCACTATGGAGTTG |
| **Construction of *Tr45852* gene deletion vector** | |
| t45852-D1 | ATTACGAATTCTTAATTAATTGCCAGACGCTGTGCCTTC |
| t45852-D2 | CATTATACGAAGTTATTCTAGACCACGAGACACGCTGCTCAA |
| t45852-D3 | ACTAGTGAGCTCATTTATTGCCATAGGCGGCTTTACAC |
| t45852-D4 | AGTGCCAAGCTTATTTACATACCGTTGCTCCCTTCTGA |
| **Verification of the *Tr45852* gene deletion mutants** | |
| t45852-CF | CAGGTATCAGGCAATCACCACC |
| t45852-CR | GACTGGGCATGTTCCGAGAGT |
| t45852-OF | TCATTCTGGCTTCTGTCGCATT |
| t45852-OR | TGGCAACAGCAGGCTTATTCTT |
| **Construction of *Tr106118* gene deletion vector** | |
| t106118-D1 | ATTACGAATTCTTAATTAACCTAACGCAACGCTACCATCAG |
| t106118-D2 | CATTATACGAAGTTATTCTAGACCGATAGCAACAGCCGATACG |
| t106118-D3 | ACTAGTGAGCTCATTTGCTGGTTTGCCTTTCTGGATTC |
| t106118-D4 | AGTGCCAAGCTTATTTGCCTGCCTGACGCCTTA |
| **Verification of the *Tr106118* gene deletion mutants** | |
| t106118-CF | AGACGGGTAAGACTGCTACCAT |
| t106118-CR | GCGTCATGGACTTTGAGGGTTC |
| t106118-OF | CTTCCGCCTCAAGATGCTCAAC |
| t106118-OR | TTCCTCGTCCTCCTCCACAGT |
| **Construction of *crz1* gene deletion vector** | |
| tcrz1-D1 | ATTACGAATTCTTAATTAAGCCTCCTCCATCATCATCATCATC |
| tcrz1-D2 | CATTATACGAAGTTATTCTAGAGCAGATTCCACAGTCAGCCTTC |
| tcrz1-D3 | ACTAGTGAGCTCATTTCTCTTCCACACCTAGCTGTCTTCT |
| tcrz1-D4 | AGTGCCAAGCTTATTTGTTCTCAATTACGGCAGTCCTTCTT |
| **Verification of the *crz1* gene deletion mutants** | |
| tcrz1-CF | CTAAGCCGTGGTCCCTTTCG |
| tcrz1-CR | TCTCCAGCCTAGATCTTGTCTCTTG |
| tcrz1-OF | TCGCACAGTCCAGCCATCAG |
| tcrz1-OR | CGGTATGAGTTCGCAGGTGAGA |
| **Construction of *pmr1* gene deletion vector** | |
| tpmr1-D1 | ATTACGAATTCTTAATTAAGCTCTCCATCCAGTGCTACCT |
| tpmr1-D2 | CATTATACGAAGTTATTCTAGATCCGAACAATCAATCAACCTCCAA |
| tpmr1-D3 | ACTAGTGAGCTCATTTAAAGGGACGGATGGGCTTCAT |
| tpmr1-D4 | AGTGCCAAGCTTATTTGATGCTGCTGCTGCTGATG |
| **Verification of the *pmr1* gene deletion mutants** | |
| tpmr1-CF | ACCTTGGGTACACCAGCATGT |
| tpmr1-CR | GAGAGGTCGTTCTGCGACTCAA |
| tpmr1-OF | GGCTCCATTGCGTCAAGTGTT |
| tpmr1-OR | AGTCTCGGATGTCGTGCTCTT |
| **Construction of *tpho84-1* gene re-complementation vector** | |
| tpho84-1-rc1 | ACTAGTGAGCTCATTTACAGATTGACCCCTCGGTTG |
| tpho84-1-rc2 | CAGAAGATGATGAGGCCAATGACATCGC |
| tpho84-1-rc3 | CCTCATCATCTTCTGGCGTGTGATTATGGGT |
| tpho84-1-rc4 | AGTGCCAAGCTTATTTCGTAACCCCATTCGAAGACCCC |
| **Construction of *tpho84-2* gene re-complementation vector** | |
| tpho84-2-rc1 | ACTAGTGAGCTCATTTCTCACGTTGTTGTCACCGTAAC |
| tpho84-2-rc2 | TGCCCACGACTTCCAGGTATGTCACACTGG |
| tpho84-2-rc3 | TGGAAGTCGTGGGCATCATGATTGGCCA |
| tpho84-2-rc4 | AGTGCCAAGCTTATTTCGGAGCCTTTGGGCGGTTT |
| **Construction of *rfp-tpho84-1* and *rfp-tpho84-2* vector** | |
| rfp-tpho84-1-F1 | GATTACGAATTCTTAATTAAGCAGGCGAATAGTGGCTTGAA |
| rfp-tpho84-1-F2 | ATTATACGAAGTTATTCTAGAGACAACAACAACAACAACAACAC |
| rfp-tpho84-1-F3 | TGGAGGTGGCGGGAGTATGGCCTTCGGAAAGACATCTGGA |
| rfp-tpho84-1-F4 | AGTGCCAAGCTTATTTGTAGTGGTGGAAGAAGTCGCTCAAG |
| rfp-tpho84-2-F1 | GATTACGAATTCTTAATTAAGTAGACAGGCAACAGGCAATCG |
| rfp-tpho84-2-F2 | ATTATACGAAGTTATTCTAGAGCGTGGATGAGGATGAGGATG |
| rfp-tpho84-2-F3 | TGGAGGTGGCGGGAGTATGGCCAGCGAAAAGCCGAC |
| rfp-tpho84-2-F4 | AGTGCCAAGCTTATTTAAGTGACGAGGTGGCGGTGAT |
| Pcbh1-1 | ACTAGTGAGCTCATTTACCTGTAAAGCCGCAATGCAGC |
| Pcbh1-2 | CATGATGCGCAGTCCGCG |
| RFP-1 | GCGGACTGCGCATCATGGCCTCCTCCGAGAACGTC |
| RFP-2 | ACTCCCGCCACCTCCACTCCCGCCACCTCCCAGGAACAGGTGGTGGCGG |
| **Verification of the *rfp-tpho84-1* and *rfp-tpho84-2* mutants** | |
| rfp-tpho84-1-CF | GGGTTTCCACGCCGTCTTTG |
| rfp-tpho84-1-CR | GGCAACGGAAACCCAGTATCC |
| rfp-tpho84-2-CF | ACTACTACAGCCGGGATCATCA |
| rfp-tpho84-2-CR | AAGCTGCTTGGAGGCGTGAG |
