# Supplementary figures and images for: Mn2+ modulates the expression of cellulase genes in Trichoderma reesei Rut-C30 via calcium signaling
Source: Biotechnol Biofuels. 2018 Mar 1;11:54. doi: 10.1186/s13068-018-1055-6 (PMC5831609; doi:10.1186/s13068-018-1055-6)

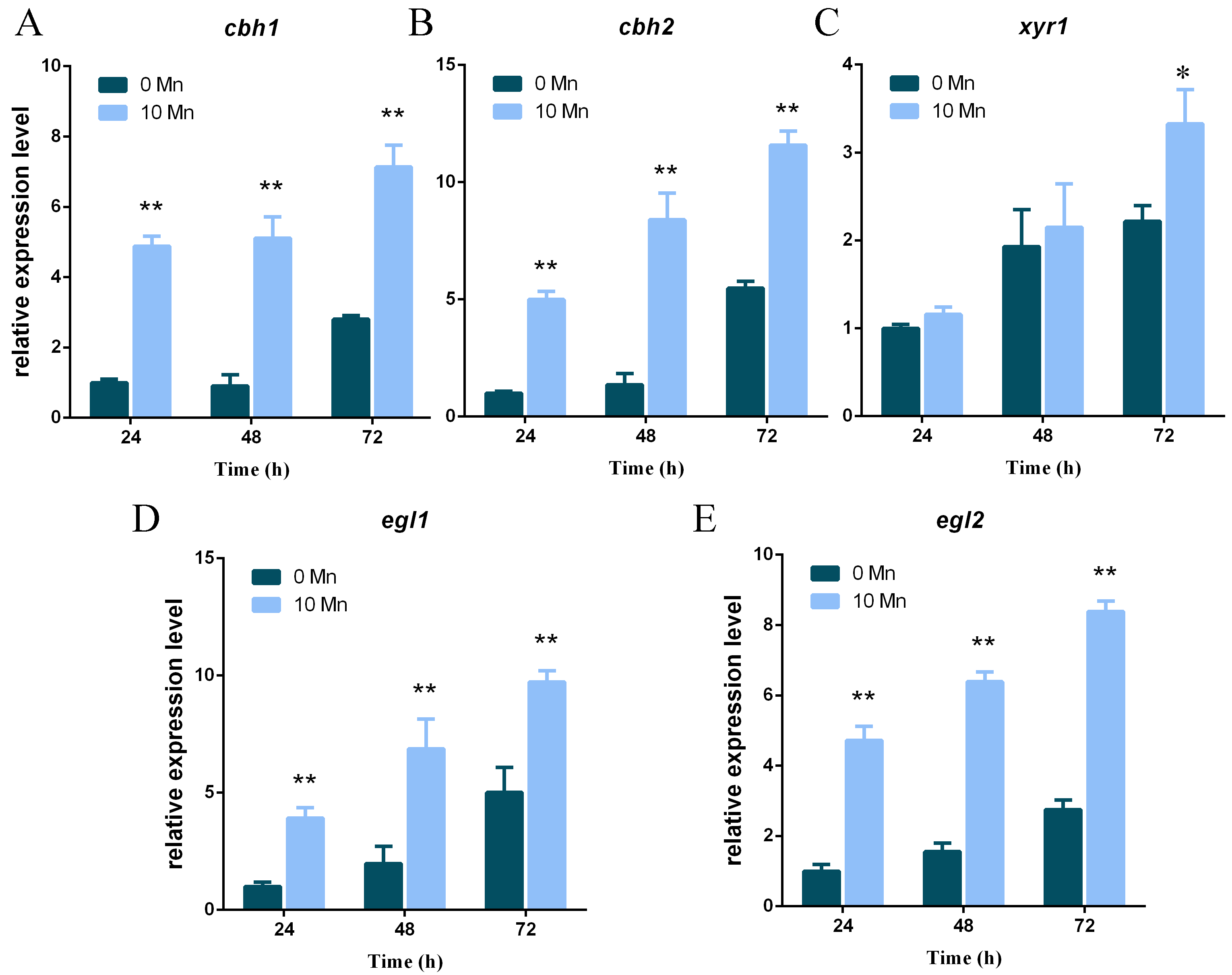

Supplement: Supplementary file 2 — Additional file 2: Figure S1. Influence of Mn2+ on the transcriptional levels of cellulase-encoding genes in T. reesei Rut-C30. A–E The effects of Mn2+ on the transcriptional levels of cbh1 (A), cbh2 (B), xyr1 (C), egl1 (D) and egl2 (E). 0 Mn, no Mn2+ was added to the medium; 10 Mn, final concentration of 10 mM. Three independent experiments with three biological replicates each were performed. Values are the means ± SD of the results from three independent experiments. Asterisks indicate significant differences from untreated strains (*p< 0.05, **p < 0.01, Student’s t test). [file 13068_2018_1055_MOESM2_ESM.tif]

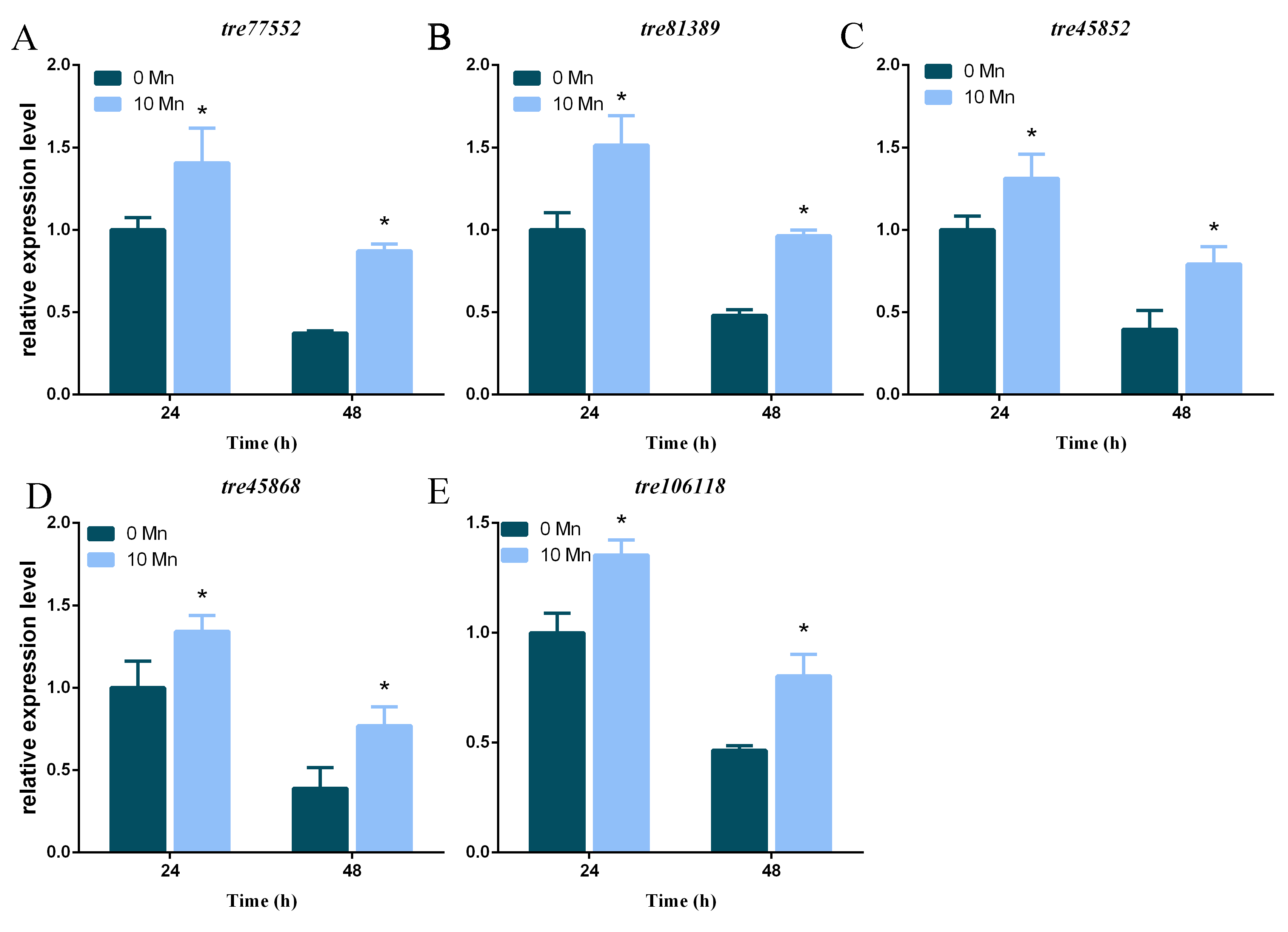

Supplement: Supplementary file 3 — Additional file 3: Figure S2. Influence of Mn2+ addition on the transcriptional levels of putative Mn2+ transport-encoding genes. A–E The effect of Mn2+ on the transcriptional levels of tre77552 (A), tre81389 (B), tre45852 (C), tre45868 (D), and tre106118 (E). 0 Mn, no Mn2+ was added to the medium; 10 Mn, final concentration of 10 mM. Values are the means ± SD of the results from three independent experiments. Asterisks indicate significant differences from untreated strains (*p< 0.05, Student’s t test). [file 13068_2018_1055_MOESM3_ESM.tif]

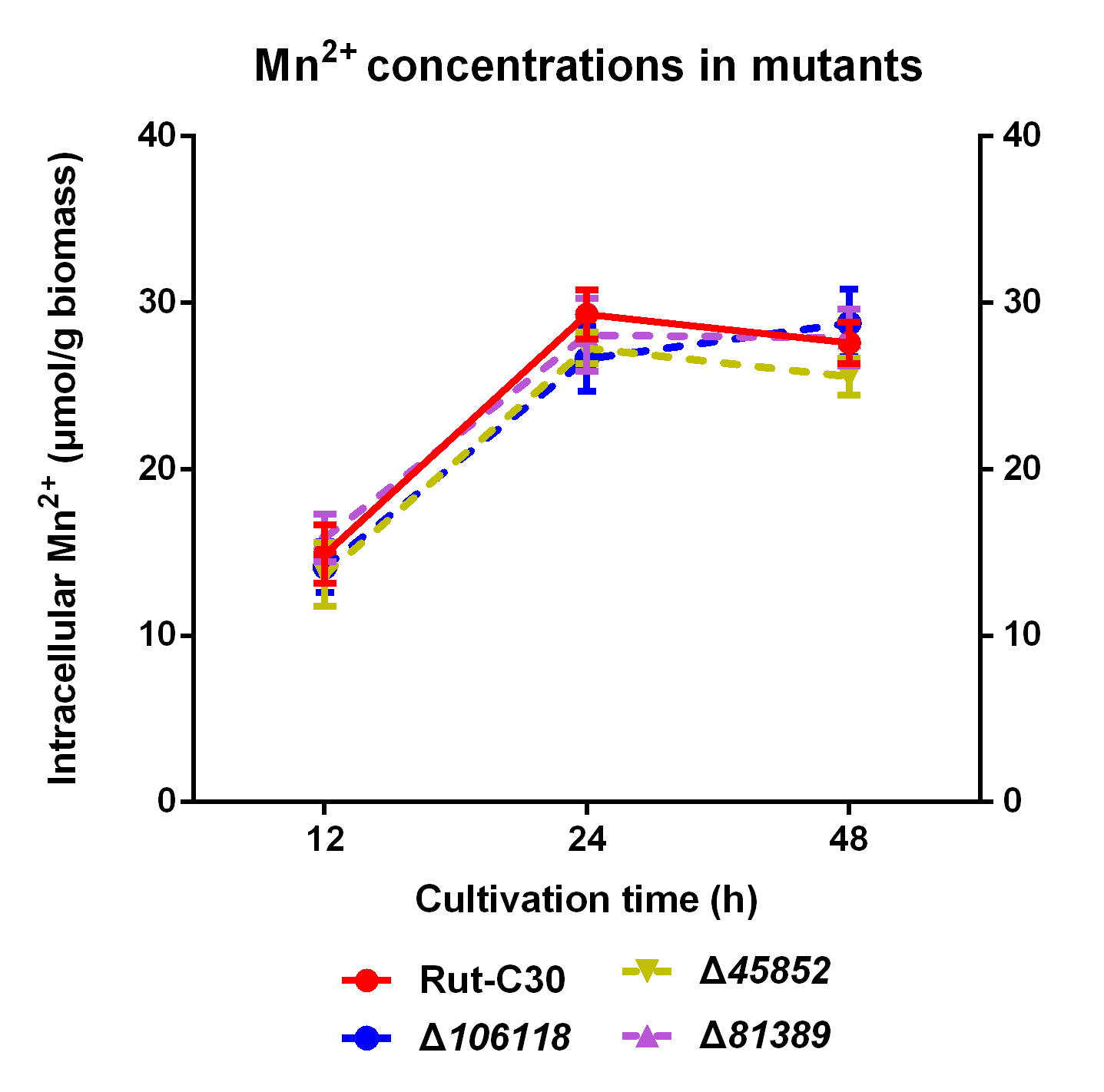

Supplement: Supplementary file 4 — Additional file 4: Figure S3. Concentrations of Mn2+ in T. reesei Rut-C30 and its derivative mutants. The concentrations of intracellular Mn2+ of T. reesei Rut-C30 and its derivative mutant strains were examined after cultured in medium containing 10 mM MnCl2. Values are the means ± SD of the results from three independent experiments. Asterisks indicate significant differences from parental strain Rut-C30 (*p< 0.05, Student’s t test). [file 13068_2018_1055_MOESM4_ESM.tif]

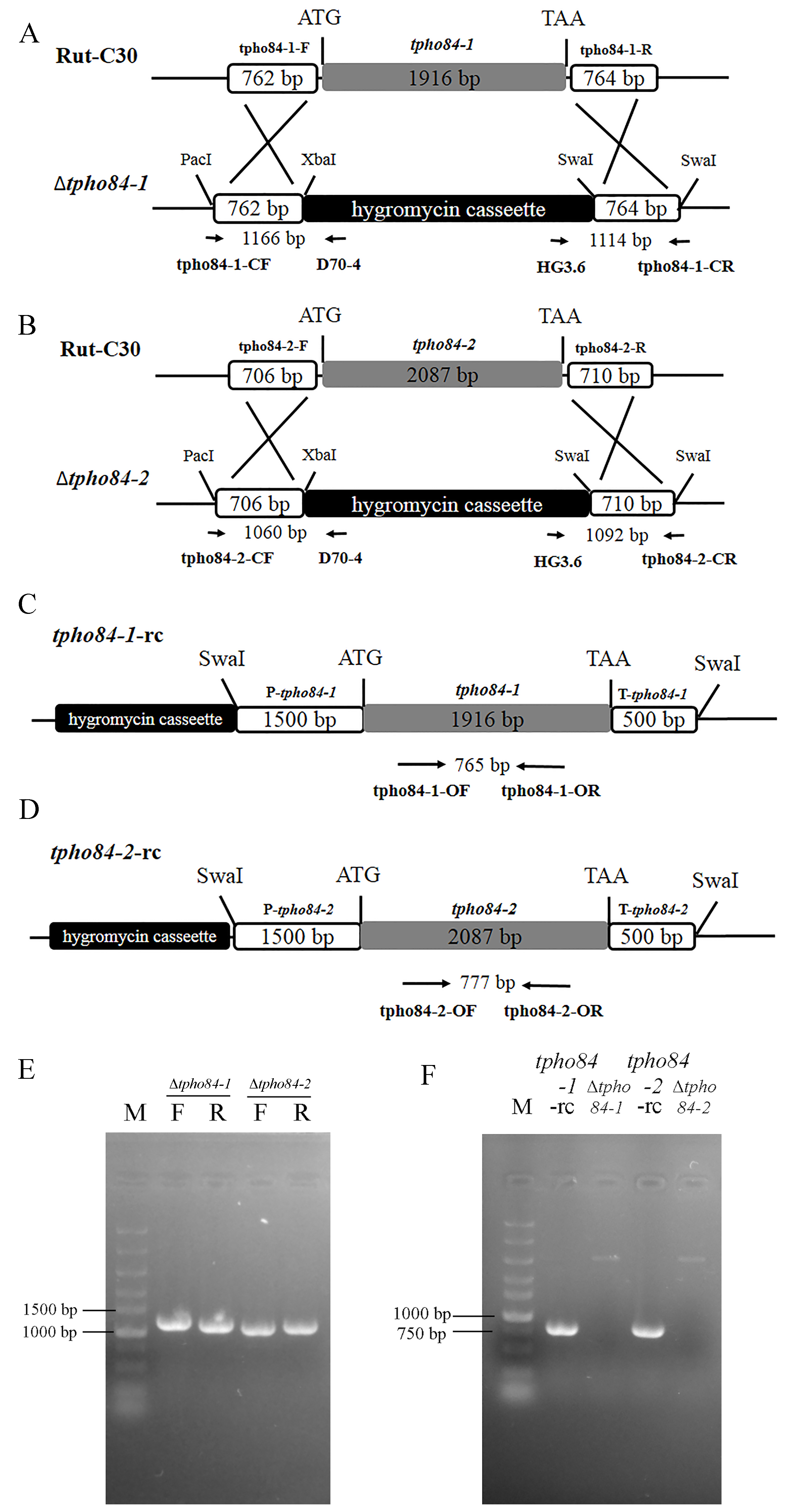

Supplement: Supplementary file 5 — Additional file 5: Figure S4. Construction and verification of Δtpho84-1, Δtpho84-2, tpho84-1-rc, tpho84-2-rc mutants. (A) Schematic representation of the tpho84-1 locus from Rut-C30 and Δtpho84-1 mutant. The binding sites of primers on the genome of Rut-C30 and Δtpho84-1, and the expected sizes of the products in PCR verification are given. The region from +1 to +1916 bp relative to the translation start site of tpho84-1 (grey box) was replaced with the hygromycin resistance expression cassette (hygromycin, black box). (B) Schematic representation of the tpho84-2 locus from Rut-C30 and Δtpho84-2 mutant. The binding sites of primers on the genome of Rut-C30 and Δtpho84-2 and the expected sizes of the PCR products are given. The region from +1 to +2087 bp relative to the translation start site of tpho84-2 (grey box) was replaced with the hygromycin resistance expression cassette (hygromycin, black box). (C) Schematic representation of the P-tpho84-1-tpho84-1-T-tpho84-1 cassette in tpho84-1-rc strains. The primer pairs indicated were used in the verification of the expression cassette. P-tpho84-1, the possible promoter of tpho84-1; T-tpho84-1, the possible terminator of tpho84-1. (D) Schematic representation of the P-tpho84-2-tpho84-2-T-tpho84-2 cassette in tpho84-2-rc strains. The primer pairs indicated were used in the verification of the expression cassette. P-tpho84-2, the possible promoter of tpho84-2; T-tpho84-2, the possible terminator of tpho84-2. (E) PCR verification of the Δtpho84-1 and Δtpho84-2 mutants. Lane M, DNA molecular mass maker. PCR amplification results of the Dtpho84-1-F were obtained using tpho84-1-CF with D70-4 and Dtpho84-1-R were obtained using HG3.6 with tpho84-1-CR (see Additional file 1: Table S1). PCR amplification results of the Dtpho84-2-F were obtained using tpho84-2-CF with D70-4 and Dtpho84-2-R were obtained using HG3.6 with tpho84-2-CR. (F) PCR verification of the tpho84-1-rc and tpho84-2-rc strains. Lane M, DNA molecular mass maker [file 13068_2018_1055_MOESM5_ESM.tif]

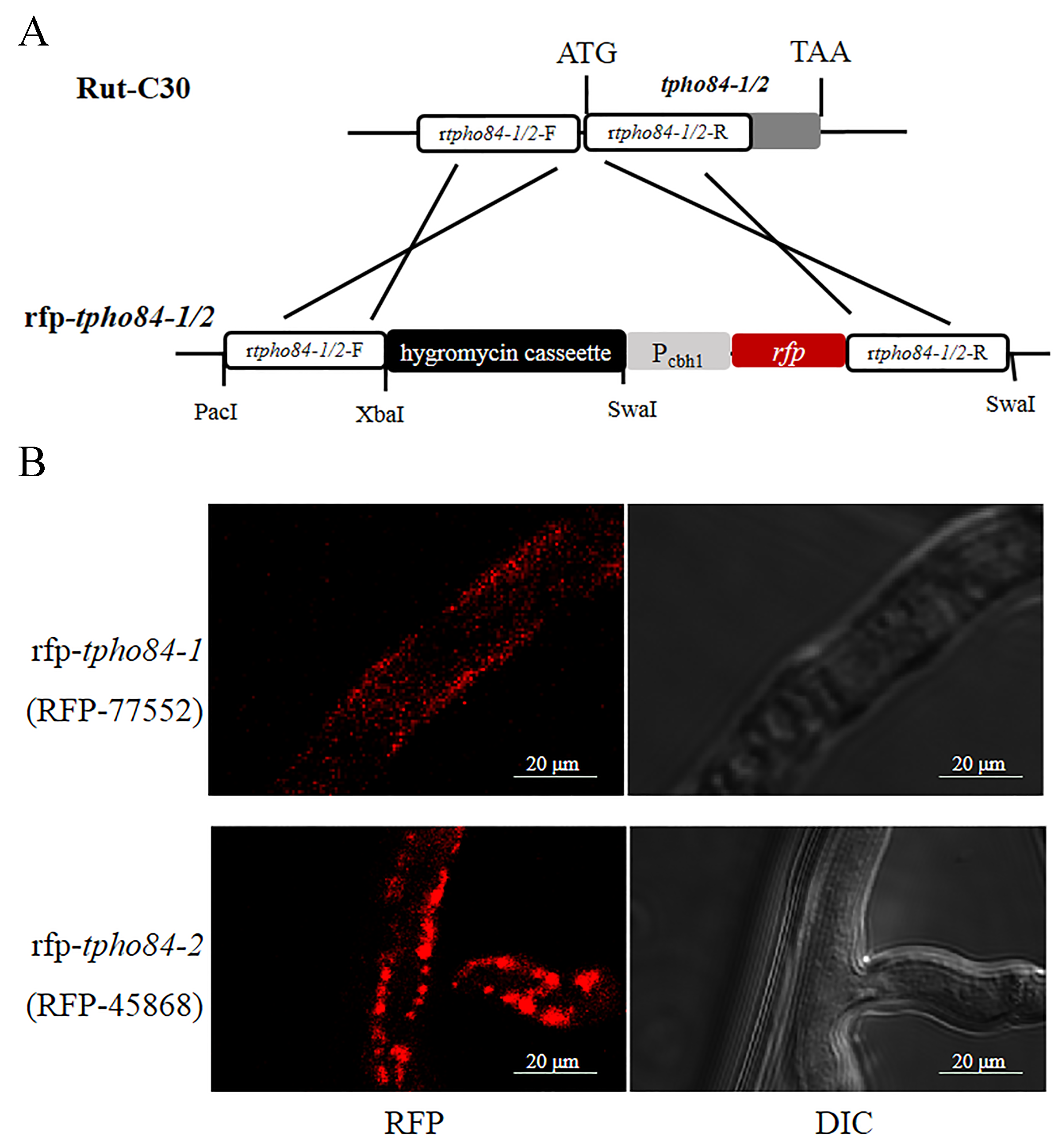

Supplement: Supplementary file 6 — Additional file 6: Figure S5. Subcellular localization of TPHO84-1 and TPHO84-2. Schematic representation of the plasmids used for expression of RFP-TPHO84-1 and RFP-TPHO84-2. RFP was fused to the N-terminal of TPHO84-1 and TPHO84-2 and expressed under the control of the cbh1 promoter. B. In vivo epifluorescence analysis of rfp-tpho84-1 and rfp-tpho84-2 transformants. The transformants were cultured in liquid Mandels’ medium for 48–60 h and observed using Laser Scanning Confocal Microscopy. [file 13068_2018_1055_MOESM6_ESM.tif]

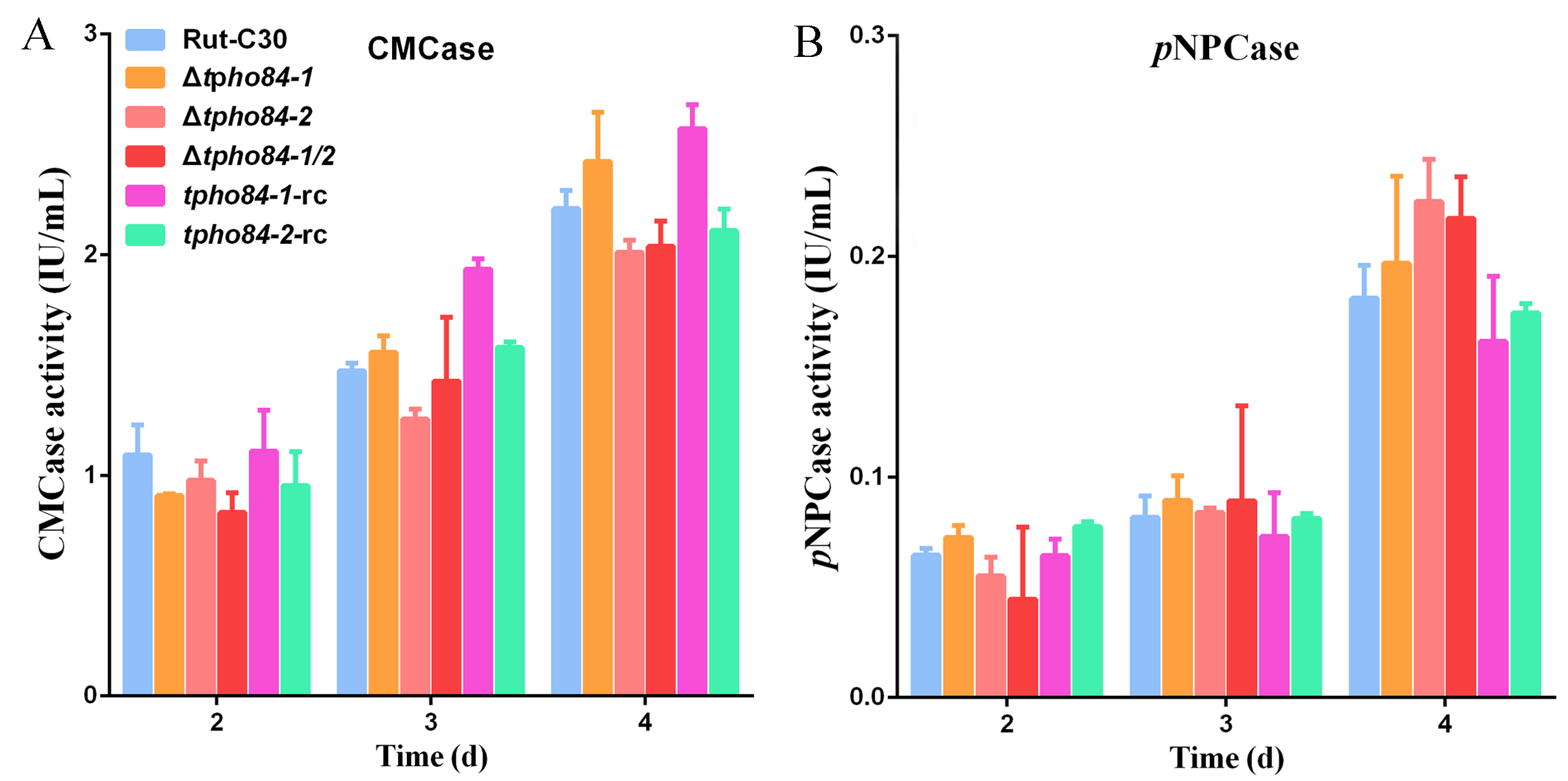

Supplement: Supplementary file 7 — Additional file 7: Figure S6. Cellulase activities of T. reesei Rut-C30 and its derivative mutant strains. The activities were examined after cultured in medium containing 0 mM Mn2+. (A) CMCase activities, (B) pNPCase activities. Values are the means ± SD of the results from three independent experiments. [file 13068_2018_1055_MOESM7_ESM.tif]

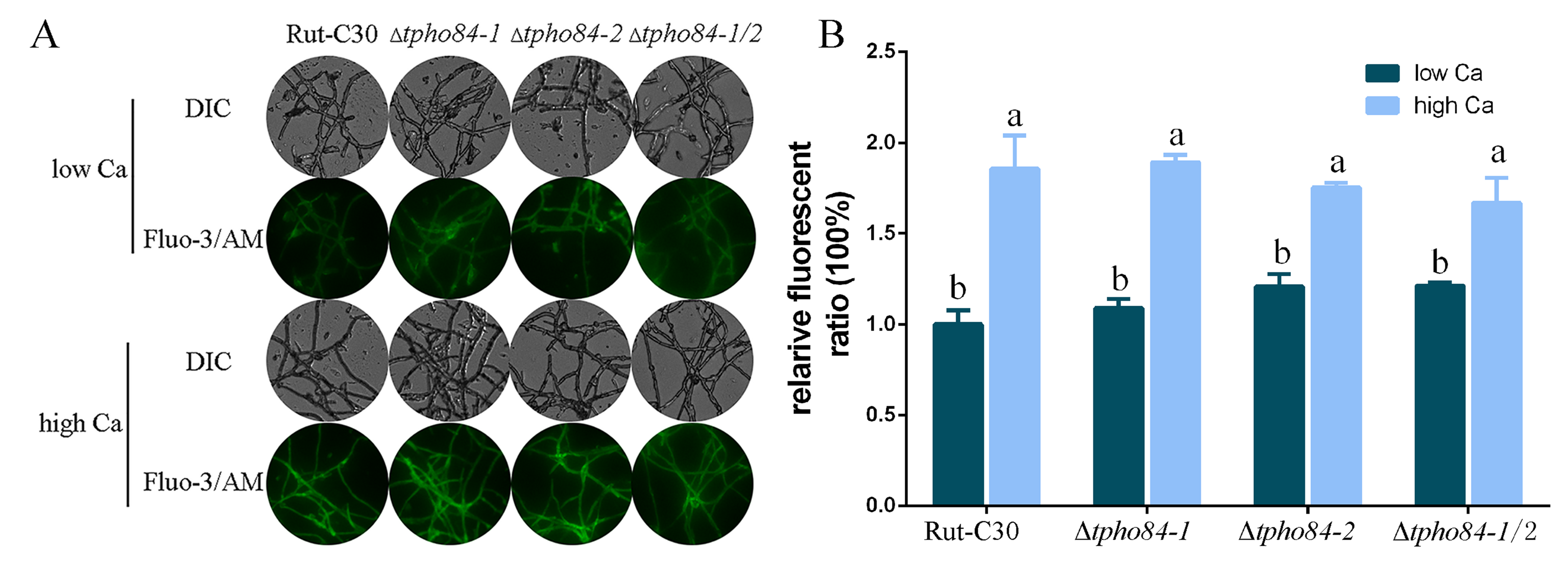

Supplement: Supplementary file 10 — Additional file 10: Figure S13. Cytosolic Ca2+ levels increase after Ca2+ addition. A. The analysis of cytosolic Ca2+ levels via a Ca2+ fluorescent probe Fluo-3/AM. The T. reesei Rut-C30 and its derivative mutant strains were cultured in liquid minimal medium (initial 5.4 mM Ca2+) for 48–60 h with extra 0 or 10 mM CaCl2 supplementation (low Ca or high Ca, respectively). For detection, 50 μM Fluo-3/AM was used, and the intensity was monitored using Automatic Inverted Fluorescence Microscopy. Green fluorescence represents the free cytosolic Ca2+. DIC, differential interference contrast. B. Comparative fluorescence ratio analysis of Ca2+ influence on cytosolic Ca2+ levels. The y-axis represents the Ca2+ fluorescence ratio measured by CLSM, and the x-axis represents the different strains tested. [file 13068_2018_1055_MOESM10_ESM.tif]

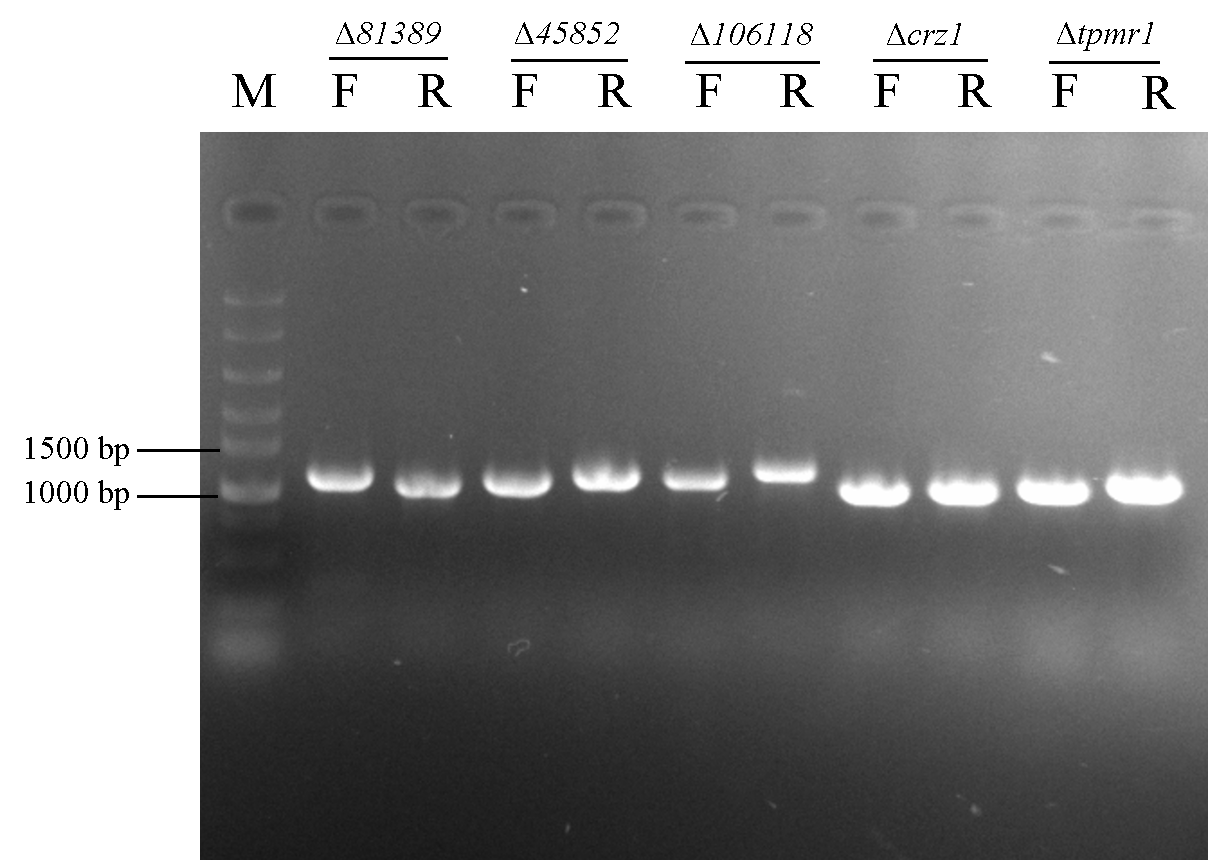

Supplement: Supplementary file 11 — Additional file 11: Figure S14. Verification of Δ81389, Δ45852, Δ106118, Δcrz1, and Δtpmr1 mutants. Lane M, DNA molecular mass maker. PCR amplification results of the F were obtained using t81389-, t45852-, t106118-, tcrz1-, and ttpmr1-CF, respectively, with D70-4, and R were obtained using HG3.6 with t81389-, t45852-, t106118-, tcrz1-, and tpmr1-CR, respectively. PCR amplification using primer pairs t81389-, t45852-, t106118-, tcrz1-, and tpmr1-OF/OR, respectively, was performed as a negative confirm for gene deletion. [file 13068_2018_1055_MOESM11_ESM.tif]

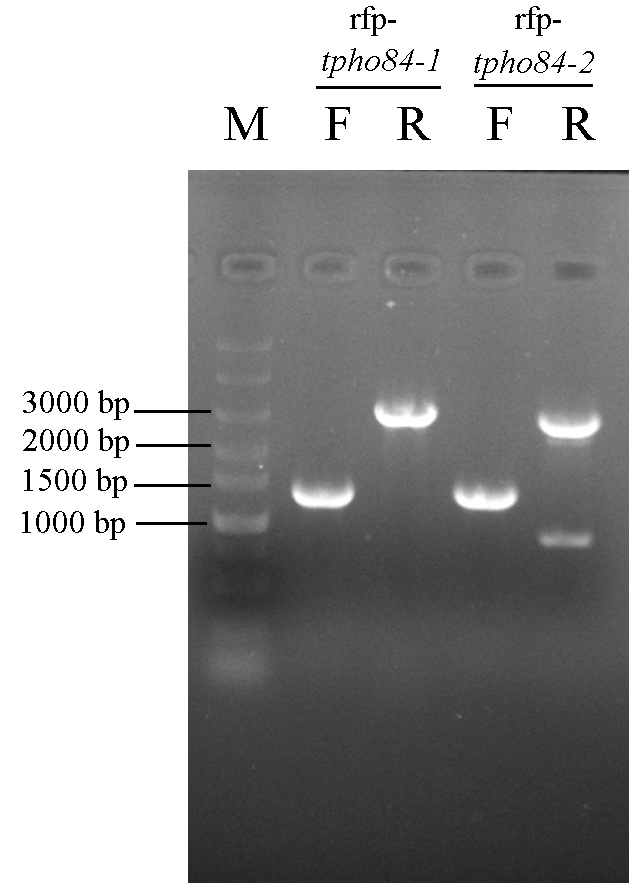

Supplement: Supplementary file 12 — Additional file 12: Figure S15. PCR Verification of rfp-tpho84-1, rfp-tpho84-2 mutants. Lane M, DNA molecular mass marker. PCR amplification results of the rfp-tpho84-1-F were obtained using rfp-tpho84-1-CF with D70-4 and rfp-tpho84-1-R were obtained using HG3.6 with rfp-tpho84-1-CR (Additional file 1: Table S1). PCR amplification results of the rfp-tpho84-2-F were obtained using rfp-tpho84-2-CF with D70-4 and rfp-tpho84-2-R were obtained using HG3.6 ith rfp-tpho84-2-CR. [file 13068_2018_1055_MOESM12_ESM.tif]
