# Supplementary material for: Mn2+ modulates the expression of cellulase genes in Trichoderma reesei Rut-C30 via calcium signaling
Source: Biotechnol Biofuels. 2018 Mar 1;11:54. doi: 10.1186/s13068-018-1055-6 (PMC5831609; doi:10.1186/s13068-018-1055-6)
Supplement: Supplementary file 8 — Additional file 8. Data for corresponding cellulase activity per biomass. The CMCase/biomass activity and pNPCase/biomass activity of T. reesei strains were examined. For every experiment, three biological replicates were performed with three technical replicates each. Values are the means ± SD of the results from three independent experiments. Asterisks indicate significant differences (*p< 0.05, Student’s t test). [file 13068_2018_1055_MOESM8_ESM.docx]

Fig. S7 (corresponds to Fig. 1C, D)


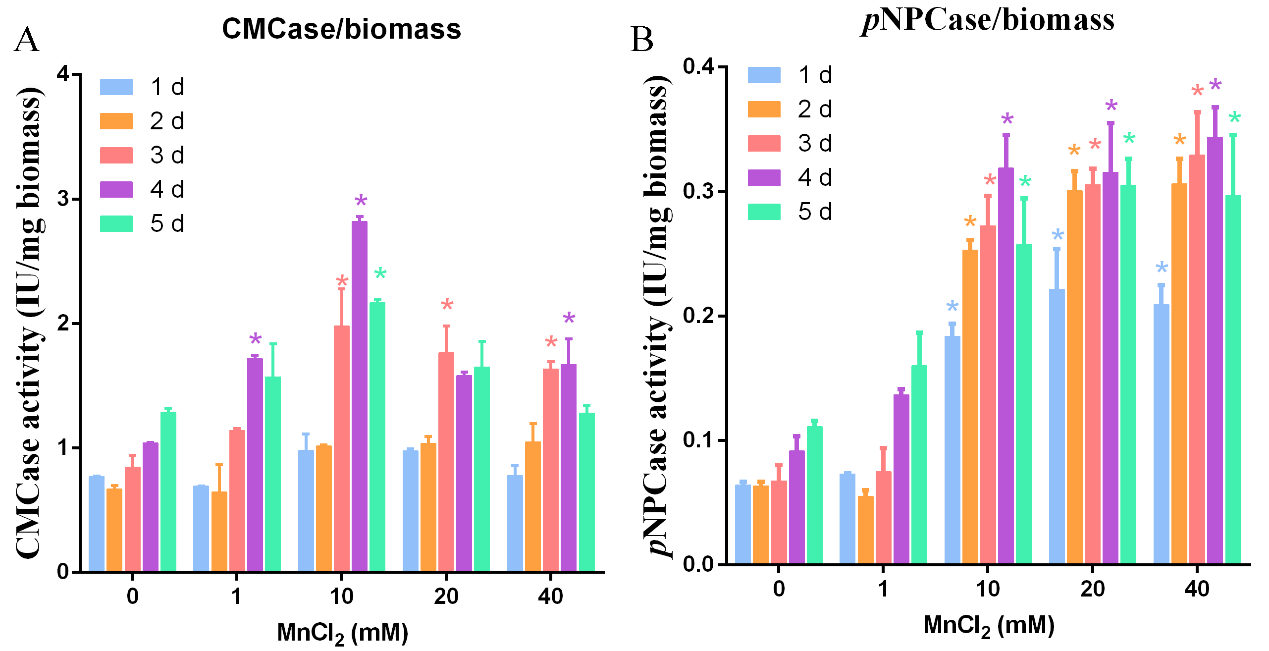


**CMCase activity per biomass**

***p*NPCase activity per biomass**

Fig. S7 **A-B.** The effects of different concentrations of Mn^2+^ (final concentration 0, 1, 10, 20 and 40 mM) on CMCase activity per biomass (A) and *p*NPCase activity per biomass (B) of *T. reesei* Rut-C30. For every experiment, three biological replicates were performed with three technical replicates each. Values are the means±SD of the results from three independent experiments. Asterisks indicate a significant difference compared to the untreated strain (*p< 0.05, Student’s *t* test).

Fig. S8 (corresponds to Fig. 3C, D)


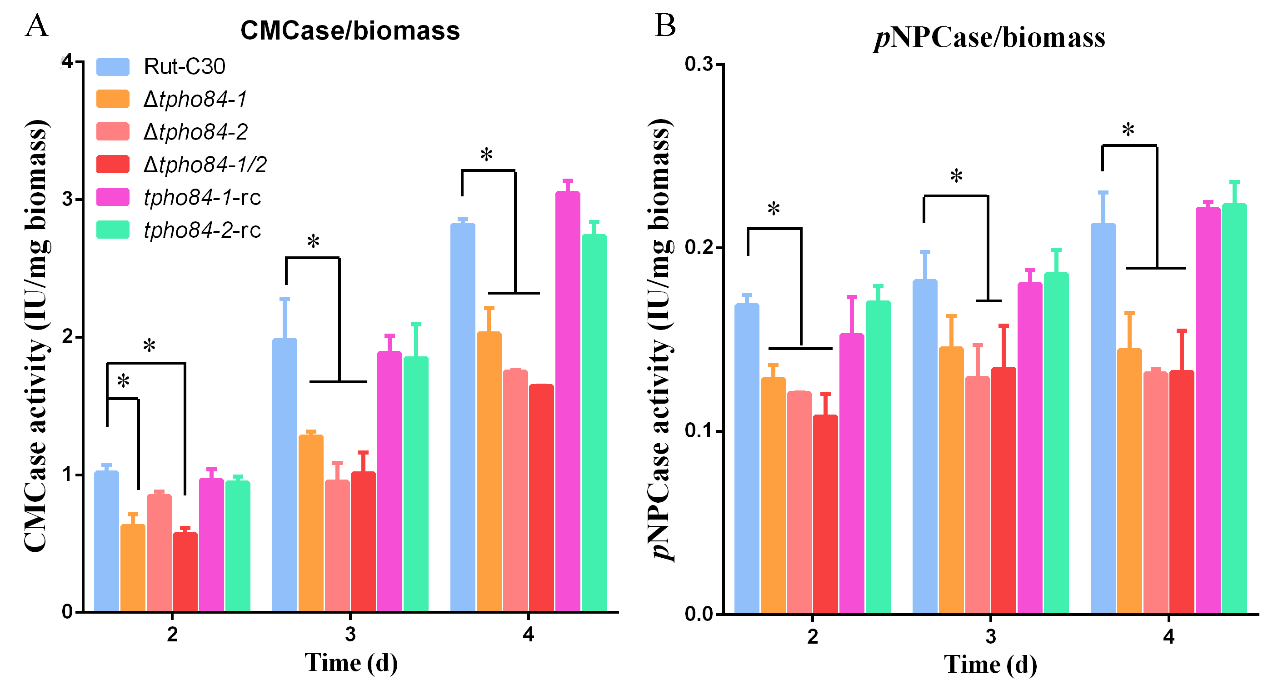


**CMCase activity per biomass**

***p*NPCase activity per biomass**

Fig. S8 **A-B.** The CMCase activity per biomass (A) and *p*NPCase activity per biomass (B) of *T. reesei* Rut-C30 and its derivative mutant strains were examined after culture in medium containing 0 or 10 mM MnCl_2_. For every experiment, three biological replicates were performed with three technical replicates each. Values are the means±SD of the results from three independent experiments. Asterisks indicate significant differences (*p< 0.05, Student’s *t* test).

Fig. S9 (corresponds to Fig. 5C, D)


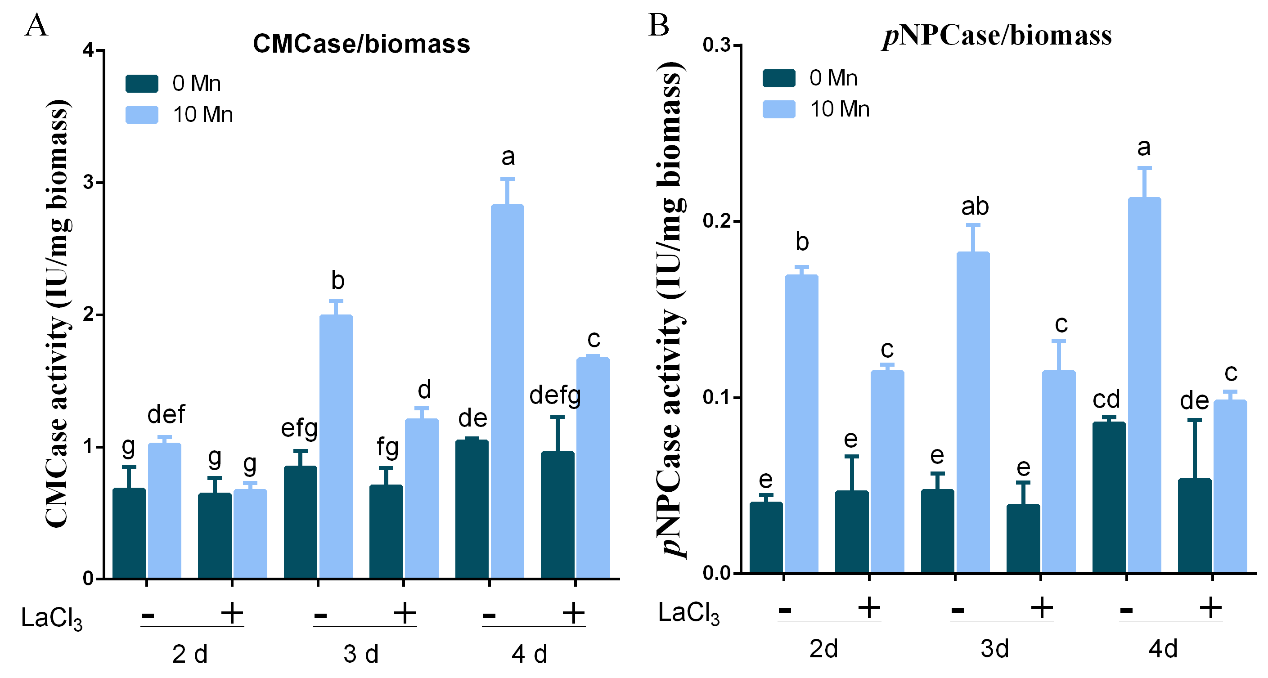


**CMCase activity per biomass**

***p*NPCase activity per biomass**

Fig. S9 **A-B.** The CMCase activity per biomass (A) and *p*NPCase activity per biomass (B) of *T. reesei* Rut-C30 were examined after culture in medium containing 0 or 10 mM MnCl_2_ and with (-) or without (+) 5 mM LaCl_3_. For every experiment, three biological replicates were performed with three technical replicates each. Values are the means±SD of the results from three independent experiments. Different letters indicate significant differences between the columns (p< 0.05, according to Duncan’s multiple-range test).

Fig. S10 (corresponds to Fig. 6A, B)


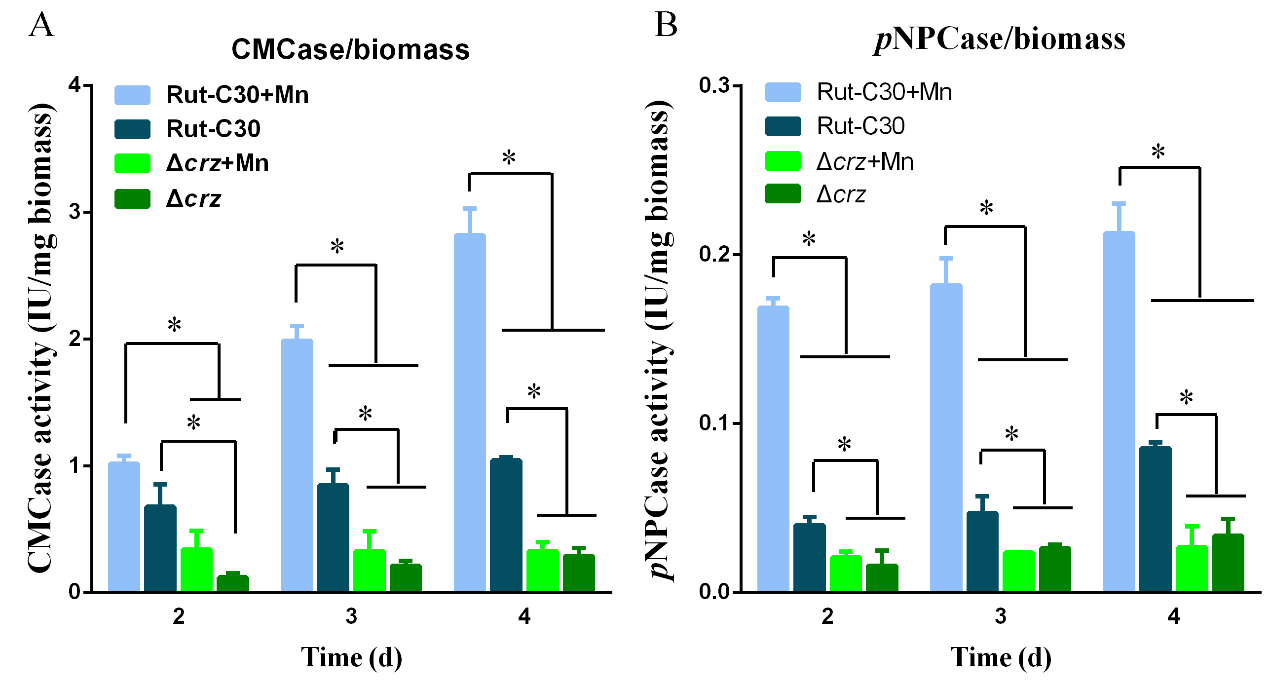


**CMCase activity per biomass**

***p*NPCase activity per biomass**

Fig. S10 **A-B.** The CMCase activity per biomass (A) and *p*NPCase activity per biomass (B) of *T. reesei* Rut-C30 and D*crz1* strains supplemented with 0 or 10 mM MnCl_2_. For every experiment, three biological replicates were performed with three technical replicates each. Asterisks indicate significant differences (*p< 0.05, Student’s *t* test).

Fig. S11 (corresponds to Fig. 7C, D)


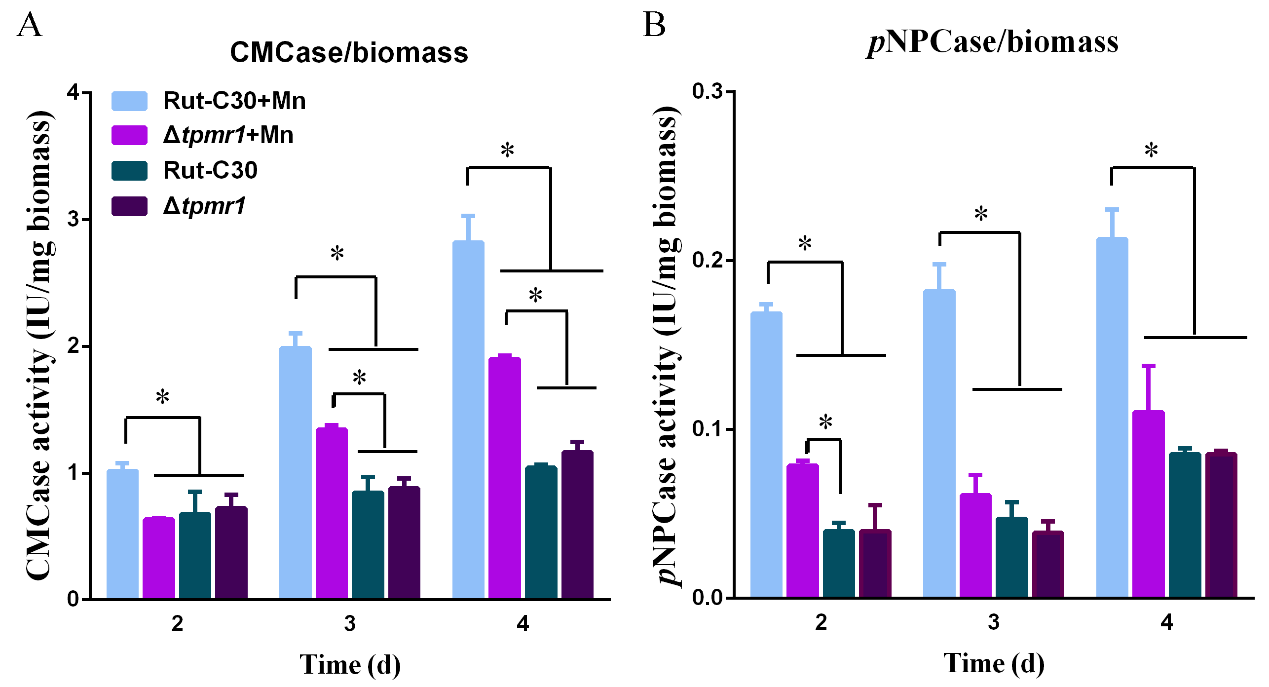


**CMCase activity per biomass**

***p*NPCase activity per biomass**

Fig. S11 **A-B.** The CMCase activity per biomass (A) and *p*NPCase activity per biomass (B) of *T. reesei* Rut-C30 and Δ*tpmr1* strains were examined after culture in medium containing 0 or 10 mM MnCl_2_. For every experiment, three biological replicates were performed with three technical replicates each. Asterisks indicate significant differences (*p< 0.05, Student’s *t* test).
